# Preferences for COVID-19 Vaccines: Systematic Literature Review of Discrete Choice Experiments

**DOI:** 10.2196/56546

**Published:** 2024-07-29

**Authors:** Yiting Huang, Shuaixin Feng, Yuyan Zhao, Haode Wang, Hongbo Jiang

**Affiliations:** 1 Department of Epidemiology and Biostatistics, School of Public Health Guangdong Pharmaceutical University Guangzhou China; 2 Department of Medical Statistics, School of Basic Medicine and Public Health Jinan University Guangzhou China; 3 Outpatient department of Baogang the First Affiliated Hospital of Guangdong Pharmaceutical University Guangzhou China; 4 School of Health and Related Research University of Sheffield Sheffield United Kingdom; 5 Institute for Global Health University College London London United Kingdom

**Keywords:** systematic review, discrete choice experiment, preference, COVID-19, vaccine

## Abstract

**Background:**

Vaccination can be viewed as comprising the most important defensive barriers to protect susceptible groups from infection. However, vaccine hesitancy for COVID-19 is widespread worldwide.

**Objective:**

We aimed to systematically review studies eliciting the COVID-19 vaccine preference using discrete choice experiments.

**Methods:**

A literature search was conducted in PubMed, Embase, Web of Science, Scopus, and CINAHL Plus platforms in April 2023. Search terms included *discrete choice experiments*, *COVID-19*, and *vaccines* and related synonyms. Descriptive statistics were used to summarize the study characteristics. Subgroup analyses were performed by factors such as high-income countries and low- and middle-income countries and study period (before, during, and after the pandemic wave). Quality appraisal was performed using the 5-item Purpose, Respondents, Explanation, Findings, and Significance checklist.

**Results:**

The search yield a total of 623 records, and 47 studies with 53 data points were finally included. Attributes were grouped into 4 categories: outcome, process, cost, and others. The vaccine effectiveness (21/53, 40%) and safety (7/53, 13%) were the most frequently reported and important attributes. Subgroup analyses showed that vaccine effectiveness was the most important attribute, although the preference varied by subgroups. Compared to high-income countries (3/29, 10%), a higher proportion of low- and middle-income countries (4/24, 17%) prioritized safety. As the pandemic progressed, the duration of protection (2/24, 8%) during the pandemic wave and COVID-19 mortality risk (5/25, 20%) after the pandemic wave emerged as 2 of the most important attributes.

**Conclusions:**

Our review revealed the critical role of vaccine effectiveness and safety in COVID-19 vaccine preference. However, it should be noticed that preference heterogeneity was observed across subpopulations and may change over time.

**Trial Registration:**

PROSPERO CRD42023422720; https://tinyurl.com/2etf7ny7

## Introduction

### Background

Although the World Health Organization has declared the end of COVID-19 as a public health emergency [1], the persistence of this disease as a global threat should not be overlooked or underestimated [2]. Vaccination has been regarded as one of the most effective strategies against COVID-19 and reduced global COVID-19 mortality, severe disease, symptomatic cases, and COVID-19 infections [2,3]. Furthermore, studies have shown that COVID-19 vaccine also had a preventive effect against post–COVID-19 condition [4-6].

Despite significant progress made with vaccination efforts, achieving high vaccination coverage remains a challenge due to disparities in vaccine distribution and vaccine hesitancy [7-9]. Disparities in vaccine distribution have been observed between different countries, with vaccination rates varying markedly between high- and low-income countries [10]. In addition, COVID-19 vaccine hesitancy has been reported across countries [11], and booster hesitancy has also become a growing concern for public health officials [12]. Vaccine hesitancy can change over time and in response to different circumstances. Notably, vaccine hesitancy tends to increase when population-level side-effect studies are released after emergency approvals [13]. These challenges underline the need for well-designed vaccination programs to ensure equitable access and high uptake.

Designing a successful vaccination program, including vaccine selection, rollout, and accessibility, is crucial [14,15]. A thorough understanding of individual needs and preferences will allow us to better tailor vaccination programs, which will facilitate the appeal and uptake of COVID-19 vaccines [16,17]. One approach increasingly used to elicit preferences for vaccines and vaccination programs is the discrete choice experiment (DCE) [18,19]. DCEs are scientific research methods that assess preferences by presenting respondents with a series of hypothetical scenarios. In these scenarios, individuals choose among different alternatives which are characterized by specific attributes. By analyzing these choices, researchers can identify the relative importance of each attribute and estimate utility functions [20,21]. DCEs provide valuable insights into decision-making processes and allow for objective evaluation of attribute-based benefits [22-24]. Published studies have been conducted to identify and review choice-based experiments that assess vaccine preferences [18,19]. However, it is important to note that the nature of various vaccines is different, and the preference for vaccines of COVID-19 was not specifically included in these studies.

### Objective

The COVID-19 vaccines were developed under emergency conditions where there were no peer-reviewed systematic reviews of DCEs on COVID-19 vaccine preference data to inform global decision-making. The diversity in COVID-19 vaccine preferences may be attributed to disparities in vaccine development and production, vaccination scheduling and management, public trust and uptake, as well as vaccine prioritization strategies across various countries and regions [25]. Moreover, new mutant variants are more likely to infect new individuals, highlighting the need for more effective booster vaccines [26,27]. This study provides empirical evidence on the development, implementation, and follow-up of the COVID-19 vaccine and provides references for vaccine decision-making of other infectious diseases.

## Methods

We conducted our review following the PRISMA (Preferred Reporting Items for Systematic Reviews and Meta-Analyses) guidelines (Multimedia Appendix 1) [28]. This study was registered in the international prospective register of systematic reviews (PROSPERO CRD42023422720).

### Search Strategy

A literature search was conducted in PubMed, Embase, Web of Science, Scopus, and CINAHL Plus platforms in April 2023. Search terms included *discrete choice experiments*, *COVID-19*, and *vaccines* and related synonyms. Further details are provided in Multimedia Appendix 2.

### Eligibility Criteria

The inclusion and exclusion criteria are detailed in Textbox 1.

**Eligibility criteria.**

**Inclusion criteria**

- Study focus: Focused on preferences for COVID-19 vaccine (product, service and distribution, policy intervention, etc)
- Article or study type: First-hand discrete choice experiment (DCE) data analysis research

**Exclusion criteria**

- Study focus: No preferences for COVID-19 vaccine reported
- Article or study type: Not DCE research; nonoriginal research (including secondary reports, systematic reviews, conference abstracts and presentations, correspondence, editorials, and commentaries); theoretical articles; protocols; book chapters; and duplicates

### Data Screening and Extraction

Two reviewers (YH and SF) independently performed a 2-stage screening process to identify eligible studies. In the first stage, titles and abstracts were screened to exclude irrelevant studies using the web-based tool Rayyan (Rayyan Systems, Inc [29]). In the second stage, full-text versions of selected papers were assessed to ensure that the inclusion criteria were met. Both reviewers compared the selected papers at each stage to ensure agreement. Any discrepancy or uncertainty between the reviewers was addressed through discussion until a consensus was reached. If not, a third (senior) reviewer (HJ) was consulted to resolve the disagreement.

The extracted data were recorded and managed in Microsoft Excel (Microsoft Corp) software. Full texts were extracted and reviewed independently by 2 authors (YH and YZ), and any disagreements were resolved by a third reviewer (HJ). Data extraction was performed for 3 specific aspects, focusing on their relevance and importance for the analysis of the DCE: (1) study information (author, publication year, study period, country, population, and sample size); (2) information on the DCE methodology (survey administration, attribute and level selection, pilot-tested, experimental study design, choice sets per respondent, options per choice set, inclusion of an opt-out option, and statistical models); and (3) information on the DCE results (number of attributes, included attributes classified into 4 categories [outcome, process, cost, and other], and the most important attribute).

Choice-based experiments use different definitions for similar attributes [19]. To address this issue, the attributes were initially grouped into 4 main categories: outcomes, process, cost, and other. The *outcomes* category encompassed the outcomes or consequences of vaccine administration, such as safety and effectiveness. The *process* category included activities related to the delivery and administration of vaccines, such as service delivery, dosing, and visits. The *cost* category focused on the financial aspects of vaccines. Any attributes that did not fit into these 3 categories were classified as *other*, such as disease risk, incentives or penalties for vaccination, vaccine advice or support, and so on. The classification of outcome, process, cost, and other attributes depended on the aim and design of the studies. It should be noted that vaccine effectiveness and safety were phrased differently in different studies. To facilitate a comparison between studies, efficacy [11,30-41], protection rate [42,43], and decreased deaths [44] were summarized as vaccine effectiveness, whereas side effects [11,26,31,35,37,40,41,43,45-61], rare but serious risks [62], and the likelihood of having a flare [62] were summarized as vaccine safety (Multimedia Appendix 3 [11,26,30-74]).

High-income countries (HICs) and low- and middle-income countries (LMICs) were classified according to the World Bank [75]. LMICs encompass low-income, lower-middle–income, and upper-middle–income countries. On the basis of previous literatures [63,76,77], we hypothesized that individuals’ preferences for vaccines may vary depending on the status of the pandemic. Therefore, we sought to explore how COVID-19 vaccine preferences differed during different study periods. To do this, we used data from the surveillance website [78] to define the pandemic periods based on daily COVID-19 cases. The first group, *before the pandemic wave*, referred to the period before the outbreak of the pandemic, when the number of incident cases was low. The second group, *during the pandemic wave*, represented the peak of the pandemic or was characterized by a rapid increase in the number of incident cases. The third group, *after the pandemic wave*, was when the number of incident cases decreased and remained low (Multimedia Appendix 4 [11,26,30-74]).

### Quality Appraisal

The 5-item Purpose, Respondents, Explanation, Findings, and Significance (PREFS) checklist, developed by Joy et al [79], is widely accepted and used to assess the reporting quality of preference studies [18,80-84]. It evaluates studies based on criteria such as the study’s purpose, respondent sampling, explanation of assessment methods, inclusion of complete response sets in the findings, and use of significance testing.

### Data Synthesis and Analysis

This review used a combination of text and summary tables to effectively convey information about the characteristics and results of the included studies. Descriptive statistics were used to summarize the study characteristics. The findings were synthesized in a narrative format, providing an overview of the included studies, highlighting the key features of the study designs, and presenting the main findings of the COVID-19 vaccine preference studies. Subgroup analyses were performed by independent factors such as HICs or LMICs and study period (before, during, and after the pandemic wave).

## Results

### Study Selection

**Figure 1:**
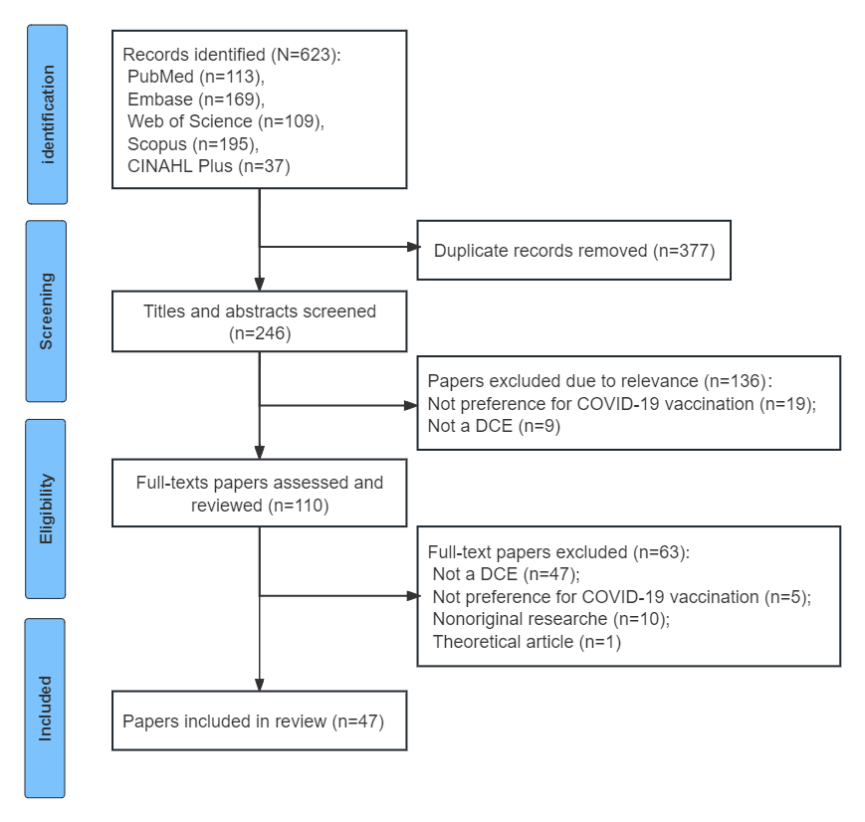
PRISMA (Preferred Reporting Items for Systematic Reviews and Meta-Analyses) flowchart of study selection process for COVID-19 vaccine preference studies using discrete choice experiments (DCEs).

The search yielded a total of 623 records. After title and abstract screening, 513 (82.3%) records were excluded. An additional 63 (10.1%) studies were excluded after full-text assessment. Finally, 47 (7.5%) studies met the eligibility criteria and were included in the review (Figure 1).

### Study and Sample Characteristics

**Figure 2:**
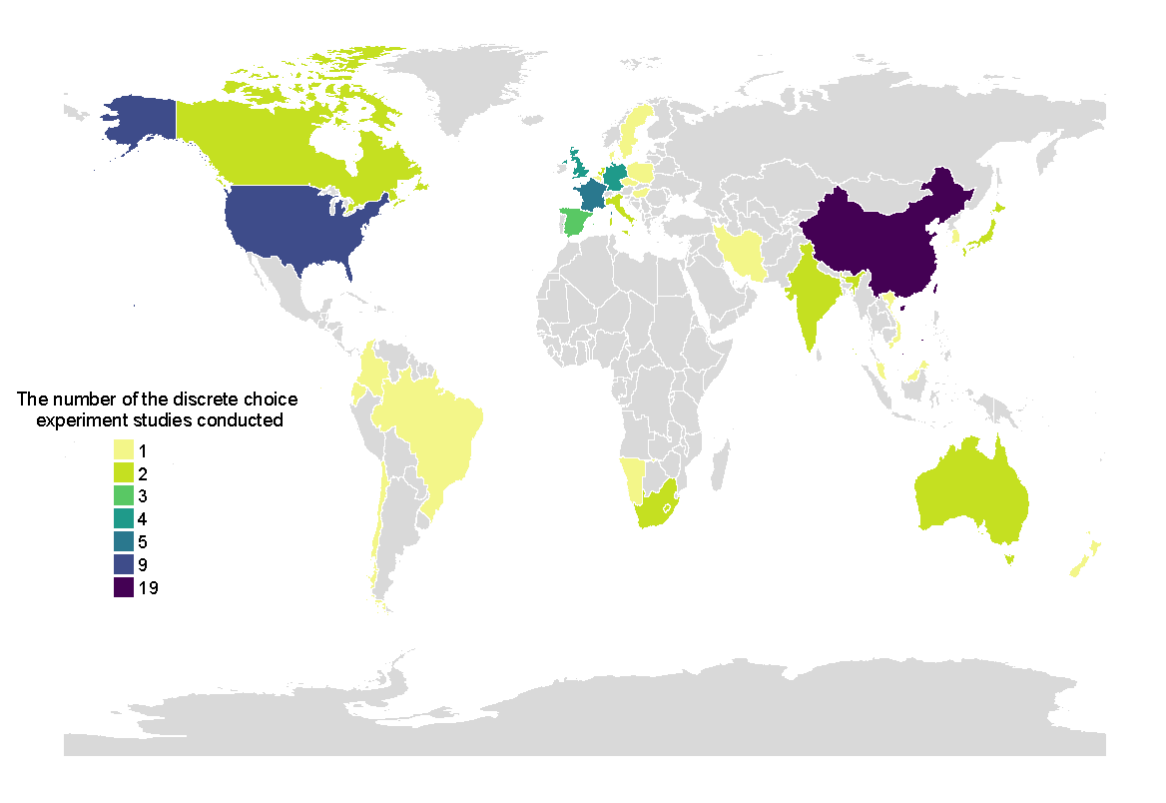
Geographical distribution of 47 included studies on COVID-19 vaccine preferences using discrete choice experiments across 29 countries.

We included 47 studies from 29 countries. Among them, 5 (11%) studies were conducted in multiple countries, with 4 studies conducted in both HICs and LMICs and 1 study conducted in >1 HICs. In addition, 22 (47%) studies were conducted in HICs, while 21 (45%) studies were conducted in LMICs. China stood out with the highest number of preference-based DCEs for COVID-19 vaccines, with 19 (40%) studies. The United States followed closely with 9 (19%) studies, followed by France (n=5, 11%), the United Kingdom (n=4, 9%), Germany (n=4, 9%), and Spain (n=3, 6%). Australia, Canada, India, Italy, Japan, the Netherlands, and South Africa had 2 (4%) studies each. All other countries had only 1 (2%) study (Figure 2). The studies were published between the years 2020 and 2023, with sample sizes ranging from 194 to 13,128 participants. The median number of participants per study was 1456 (IQR 872-2109).

Most participants were adults, although the specific focus varied. Most studies (36/47, 77%) involved general population samples, whereas some studies (11/47, 23%) included specific groups of participants. These included 5 studies conducted in universities using web-based tools, including 3 studies with university students and 2 studies with both students and staff. In addition, 3 studies involved health care workers (Chinese intensive care unit clinicians, health care workers, and health care and welfare workers); 2 studies involved parents with children aged <18 years, and 1 study involved people with chronic immune-mediated inflammatory diseases (Table 1).

**Table 1:** Characteristics of 47 included studies on COVID-19 vaccine preferences using discrete choice experiments.

Author, year	Study period	Country	Population	Sample size, n
Asim et al [32], 2023	February 26 to April 26, 2021	China	Adults	208
Bansal et al [26], 2022	May to June, 2021	India	Adults	1371
Blaga et al [50], 2023	March to September, 2021	Hungary	General population	1011
Borriello et al [56], 2021	March 27 to 31, 2020	Australia	General population	2136
Bughin et al [70], 2023	January 25 to 28, 2021	Germany	General population	1556
Chen et al [47], 2023	January 24 to March 10, 2021	China	Middle-aged and older adults aged ≥50 years	293
Chen et al [69], 2021	January 5 to 12, 2021	China	Adults	1066
Craig [60], 2021	November 9 to 11, 2020	The United States	Adults	1153
Darrudi et al [57], 2022	March 21 to July 6, 2021	Iran	Adults	685
Daziano [46], 2022	October 22 to November 24, 2020	The United States	Adults	2723
Díaz Luévano et al [39], 2021	December 18, 2020, to February 1, 2021	France	Health care and welfare workers	4346
Dong et al [66], 2020	June to July, 2020	China	Adults	1236
Dong et al [45], 2022	January 29 to February 13, 2021	India, the United Kingdom, Germany, Italy, and Spain	Adults	812
Donin et al [11], 2022	March 22 to May 3, 2021	Czech Republic	University students	445
Eshun-Wilson et al [71], 2021	March 15 to March 22, 2021	United States	General population	2985
Fu et al [30], 2020	March 17 to 18, 2020	China	Health care workers	541
Fung et al [33], 2022	July 20 to September 21, 2021	China	University students and staff members	3423
George et al [64], 2022	November 18 to December 24, 2021	South Africa	University students and staff members	1836
Hazlewood et al [62], 2023	May to August, 2021	Canada	People with chronic immune-mediated inflammatory diseases	551
Hess et al [54], 2022	Summer 2020 to the start of March 2021	Africa: Namibia, South Africa; Asia: China Japan, and South Korea; Europe: Denmark, France, Germany, Spain, and the Kingdom; North America: the United States; Oceania: Australia and New Zealand; and South America: Brazil, Chile, Colombia, and Ecuador	General population	13,128
Huang et al [48], 2021	March 24 to April 10, 2021	China	Chinese ICU^a^ clinicians	11,951
Igarashi et al [38], 2022	November 19 to 27, 2020	Japan	General population	2155
Krueger and Daziano [58], 2022	March 4 to 10, 2021	The United States	General population	1421
Leng et al [51], 2021	NR^b^	China	Adults	1883
Li et al [74], 2021	January 25 to February 25, 2021	China	University students	194
Li et al [41], 2023	January 28 to February 27, 2021	China and the United States	Middle-aged and older adult population (aged ≥41 years)	3444
Liu et al [31], 2021	January 29 to February 13, 2021	China and the United States	General population	2480
Luyten et al [72], 2022	October 6 to 16, 2020	Belgium	Adults	1944
McPhedran et al [73], 2022	March 25 to April 2, 2021	The United Kingdom	Adults	2012
McPhedran et al [42], 2021	August 27 to September 3, 2020	The United Kingdom	General population	1501
Morillon and Poder [65], 2022	October 19 to November 17, 2020	Canada	Adults	1599
Mouter et al [43], 2022	November 4 to 10, 2020	The Netherlands	General population	895
Mouter et al [44], 2022	December 1 to 4, 2020	The Netherlands	Adults	747
Panchalingam and Shi [68], 2022	October to November, 2021	United States	Parents with children aged <18 years	1456
Prosser et al [49], 2023	May 21 to June 9, 2021	The United States	Adults	1040
Schwarzinger et al [34], 2021	June 22 to July 3, 2020	France	Working-age population (aged 18-64 years)	1942
Steinert et al [63], 2022	Germany in April 2021; France, Italy, Poland, Spain, and Sweden in June 2021	France, Germany, Italy, Poland, Spain, and Sweden	Adults	6030
Teh et al [53], 2022	March 2021	Malaysia	Adults	2028
Tran et al [55], 2023	April to August, 2022	Vietnam	Adults	871
Velardo et al [40], 2021	November 30 to December 16, 2020	France	Working-age population (aged 18-64 years)	5519
Wang et al [61], 2022	August 2020	China	Adults	873
Wang et al [36], 2021	February 26 to 28, 2021	China	Working-age population (aged 18-64 years)	1773
Wang et al [35], 2022	Mid-September to the end of October, 2021	China	Parents with children <18 years old	298
Wang et al [59], 2022	May 2021	China	University students	1138
Wang et al [52], 2022	May to June, 2021	China	Adults	849
Xiao et al [67], 2022	January 28 to 31, 2021	China	Adults	1576
Zhang et al [37], 2022	July 15 to August 10, 2021	China	Adults	1200

^a^ICU: intensive care unit.

^b^NR: not reported.

### The Implementation of DCEs

Among these 47 studies, researchers commonly used a multifaceted approach to identify and select attributes and levels. Among the studies reviewed, 23 (49%) studies reported a literature review with qualitative assessments such as expert interviews and public surveys. A total of 25 (53%) studies reported a pilot DCE survey. In terms of survey administration, most studies (40/47, 85%) reported that the DCE was conducted through web-based surveys (Table 2).

**Table 2:** Conduct of 47 included studies on COVID-19 vaccine preferences using discrete choice experiments (DCEs).

Author, year	Survey administration	Attributes and levels selection	Pilot-tested DCE	Experimental study design	Choice sets per respondent	Options per choice set	Statistical models
Asim et al [32], 2023	Web based	Focus group	Yes	D-optimal algorithm design	8	2+opt out	Latent class logit model and nested logistic model
Bansal et al [26], 2022	Web based	Literature review	NR^a^	D-efficient design	6	2	Conditional logit model and nonparametric logit mixed logit model
Blaga et al [50], 2023	NR	Focus group and expert interviews	Yes	D-efficient design	8	3+opt out	Latent variable models, random parameters logit model, and hybrid random parameters logit model
Borriello et al [56], 2021	Web based	Literature review and judgment of respondent understanding and plausibility	NR	Bayesian d-efficient design	8	3+opt out	Latent class model
Bughin et al [70], 2023	Web based	On the basis of the purpose of the research and necessary calibration of the conjoint	NR	NR	10	3	Hierarchical multinomial logit model
Chen et al [47], 2023	NR	Literature review, expert interviews, and current COVID-19 vaccine development progress	Yes	Orthogonal design	12	2	Multinomial logistic regression model
Chen et al [69], 2021	Web based	Literature review	NR	D-efficient design	16	2	Conditional logit model and panel mixed logit model
Craig [60], 2021	Web based	Literature review, expert interviews, and the CDC^b^ interim playbook version 2.0	Yes	NR	8	3+opt out	Conditional logit model, latent class model, and opt-out inflated logit model
Darrudi et al [57], 2022	Web based	Literature review and expert interviews	Yes	D-efficient design	Group 1:9 and group 2:10	Group 1: 2 and group 2: 2	Conditional logit model
Daziano [46], 2022	Web based	Literature review and focus group	Yes	Bayesian efficient design	7	2+opt out	Latent class logit model, conditional logit model, and random effects logit model
Díaz Luévano et al [39], 2021	Web based	Literature review	Yes	Efficient design	8	1+opt out	Random intercept logit models
Dong et al [66], 2020	Web based	Literature review, expert interviews, and public interviews	Yes	D-optimal algorithm design	10+validity	2	Mixed logit regression model
Dong et al [45], 2022	Web based	NR	Yes	NR	NR	NR	Conditional logit model
Donin et al [11], 2022	Web based	Literature review	Yes	D-efficient design	NR	2+opt out	Hierarchical Bayes
Eshun-Wilson et al [71], 2021	Web based	Expert interviews, expert discussion, and literature review	Yes	Fractional factorial design	10	2+opt out	Mixed logit model and latent class model
Fu et al [30], 2020	Web based	Literature review, focus group, and expert interviews	Yes	Fractional factorial design	8+ validity	2	Binary logistic regression model
Fung et al [33], 2022	Web based	Literature review and expert interviews	NR	Orthogonal design	8	2+opt out	Mixed logit model
George et al [64], 2022	Web based	Literature review and a series of meetings and discussions with the study team and key stakeholders at UKZN^c^	NR	Fractional factorial design	8	2	Mixed effects logit model
Hazlewood et al [62], 2023	Web based	Guideline panel discussion	Yes	Fractional factorial design	10	2+opt out	Main-effects multinomial logit model
Hess et al [54], 2022	Web based	NR	NR	D-efficient design	6	4+opt out	Ordered logit model, latent class model, and nested logit
Huang et al [48], 2021	Web based	Expert interviews	Yes	Fractional factorial design	4	2	Multivariable conditional logistic regression model
Igarashi et al [38], 2022	Web based	Literature review	NR	Orthogonal design	12	2+opt out	Panel logit model
Krueger and Daziano [58], 2022	NR	Literature review and focus group	NR	Bayesian efficient design	7	2+opt out	Normal error components mixed logit model
Leng et al [51], 2021	Face to face	Literature review	Yes	D-efficient partial profile design	8	2	Conditional logit model
Li et al [74], 2021	Web based	NR	NR	Orthogonal design	6	2	Conditional logit model
Li et al [41], 2023	Web based	Literature review and expert interviews	NR	Fractional factorial design	13	2+opt out	Conditional logit model
Liu et al [31], 2021	Web based	Literature review and expert interviews	Yes	NR	NR	2	Conditional logit model
Luyten et al [72], 2022	Web based	Literature review	Yes	Bayesian d-optimal design	10+ validity	2	Panel mixed logit model
McPhedran et al [73], 2022	Web based	Literature review	NR	D-optimal fractional factorial design	6	2+opt out	Mixed logit model
McPhedran et al [42], 2021	Web based	Literature review	NR	Rotation design	6	2+opt out	Clustered conditional logit model and hybrid logit model
Morillon and Poder [65], 2022	Web based	Literature review, expert interviews, and public interviews	NR	Orthogonal design	11+ validity	2+opt out	Mixed logit model, latent class logit model, and multinomial logistic regression
Mouter et al [43], 2022	Web based	Literature review, expert consultations, and feedback	Yes	Bayesian d-efficient design	8	2	Panel mixed logit model
Mouter et al [44], 2022	Web based	Literature review, expert discussion, and pretest	Yes	Bayesian d-optimal design	9	2	Panel mixed logit model
Panchalingam and Shi [68], 2022	Web based	Literature review	NR	D-efficient design	10+ validity	2+opt out	Logistic regressions model and random parameter logit regressions model
Prosser et al [49], 2023	Web based	Literature review and public interviews	NR	Fractional factorial design	6	2+opt out	Bayesian logit regression and latent class analyses
Schwarzinger et al [34], 2021	Web based	Literature review and expert interviews	NR	D-efficient design	8	2+opt out	Conditional logit model
Steinert et al [63], 2022	Web based	NR	NR	D-efficient design	8	2	Conditional logit model, and fixed-effects model
Teh et al [53], 2022	Web based	Literature review, expert interviews, and focus group	Yes	Bayesian d-optimal design	10+ validity	2+opt out	Mixed logit model，and nested logit model
Tran et al [55], 2023	Web based	Literature review and expert interviews	Nr	NR	7	2	Hierarchical Bayes
Velardo et al [40], 2021	Web based	NR	NR	D-efficient design	8	2+opt out	Conditional logit model
Wang et al [61], 2022	Web based	Expert interviews and public interviews	Yes	D-efficient design	6	2+opt out	Multinominal mixed effects logit model
Wang et al [36], 2021	Web based	Individual interviews	Yes	D-optimal algorithm design	8	2+opt out	Multiple logistic regression model, nested logistic model, and separate logistic model
Wang et al [35], 2022	Web based	Literature review, qualitative interview and background information, and levels of the attributes	Yes	D-efficient design	8	2+opt out	Multiple logistic model and mixed logit model
Wang et al [59], 2022	Face to face	Literature review	NR	D-efficient partial profile design	8+ validity	2	Conditional logit model
Wang et al [52], 2022	Face to face	Literature review and expert interviews	Yes	D-efficient partial profile design	8	2	Conditional logit model, mixed logit model, and latent class model
Xiao et al [67], 2022	Web based	Literature review, research team discussions, official report, expert discussion, and pretest	Yes	Full factorial design	4	2+opt out	Random parameter logit model and constrained latent class model
Zhang et al [37], 2022	NR	Literature review, expert interviews, and several vaccines on the market	NR	Fractional factorial design	11	2+opt out	Conditional logit model

^a^NR: not reported.

^b^CDC: Center for disease control and prevention.

^c^UKZN: the University of KwaZulu-Natal.

### Attributes in DCE Studies

Of the 286 attributes identified in the 47 studies, 126 (44.1%) were categorized as *outcome* attributes, followed by 82 (28.7%) as *process* attributes, and 22 (7.7%) as *cost* attributes. The remaining 55 (19.2%) attributes were categorized as *other* attributes (Table 3 and Multimedia Appendix 3).

**Table 3:** Attributes included in 47 studies on COVID-19 vaccine preferences using discrete choice experiments.

Author, year	Attributes, n	Outcome	Process	Cost	Other	Most important attribute
Asim et al [32], 2023	7	Efficacy^a^ and safety^a^	Venue for vaccination^a^ and vaccine brand^a^	—^b^	Exemption of quarantine for vaccinated travelers^a^, uptake of recommendations from professionals, and vaccine by people around	Brand
Bansal et al [26], 2022	7	Effectiveness of vaccine^a^, side effects^a^, and duration of protection offered by the vaccine^a^	Developer^a^, and place where vaccination is administered^a^	Out-of-pocket cost^a^	The proportion of friends and family members who have taken the vaccine^a^	Vaccinated friends or family
Blaga et al [50], 2023	4	Effectiveness of the vaccine^a^, type of possible side effects^a^, and duration of protection provided by the vaccine^a^	Country of origin^a^	—	—	Duration of protection
Borriello et al [56], 2021	7	Effectiveness^a^, mild side effects^a^, and major side effects^a^	Mode of administration^a^, location^a^, and time period when the vaccine was available^a^	Cost^a^	—	Safety
Bughin et al [70], 2023	5	Effectiveness^a^	Time of COVID-19 vaccination^a^		Work site^a^, restriction level^a^, choices to get vaccinated^a^, and advantages or penalties^a^	Time of COVID-19 vaccination
Chen et al [47], 2023	5	Risk of adverse effects^a^, protective duration^a^, and effectiveness^a^	Injection doses^a^ and injection period^a^	—	—	Safety
Chen et al [69], 2021	5	Protection rate ^a^, adverse effect ^a^, and protection duration^a^	Convenience of vaccination^a^	Cost of the vaccine^a^	—	Safety
Craig [60], 2021	5	Duration of immunity ^a^, risk of severe side effects ^a^, and vaccine effectiveness^a^	Vaccination setting^a^	—	Proof of vaccination^a^	Effectiveness
Darrudi et al [57], 2022	6	Group 1: effectiveness ^a^, risk of severe complications ^a^, and duration of protection^a^	Group 1: location of vaccine production^a^; group 2: age	Group 1: price^a^; group 2: cost to the community^a^	Group 1: underlying disease^a^, employment in the health sector ^a^, potential capacity to spread the virus (virus spread)^a^, and the necessary job for society^a^	Group 1: effectiveness; group 2: potential capacity to spread the virus
Daziano [46], 2022	9	Effectiveness ^a^, days for antibodies to develop^a^, duration of protection^a^, number of people out of 10 with mild side effects ^a^, and the number of people out of 1,000,000 with severe side effects^a^	Country where vaccine was developed^a^ and introduced (months)^a^	Out-of-pocket cost^a^	Who recommends this specific vaccine^a^	Recommenders
Díaz Luévano et al [39], 2021	5	Efficacy^a^, indirect protection^a^, safety^a^, and protection duration^a^	—	—	Recommendation or incentive source^a^	Effectiveness
Dong et al [66], 2020	6	Effectiveness^a^, duration of protection^a^, and adverse event^a^	The total number of injections^a^ and origin of the product^a^	Price (Chinese Yuan)^a^	—	Effectiveness
Dong et al [45], 2022	6	Adverse effects^a^, efficacy ^a^, duration of the vaccine ^a^, and time taken for the vaccine to work^a^	Vaccine types	The cost of vaccination^a^	—	Effectiveness
Donin et al [11], 2022	6	Protection duration^a^, efficacy^a^, and risk of mild side effects^a^	Route of vaccination^a^ and travel time to vaccination site^a^	—	Recommender of the vaccine^a^	Protection duration
Eshun-Wilson et al [71], 2021	7	—	Vaccine frequency, waiting time at vaccination site, vaccination location, number of doses required per vaccination episode, and vaccination appointment scheduling	—	Vaccination enforcement and who has already received the vaccine in your community?	Vaccine frequency
Fu et al [30], 2020	7	Vaccine safety^a^ and vaccine efficacy^a^	—	Out-of-pocket costs^a^	Infection probability^a^, case fatality ratio^a^, possible trends of the epidemic^a^, and acceptance of social contacts^a^	Possible trends of the epidemic
Fung et al [33], 2022	7	Risk of a mild or moderate adverse event after vaccination^a^, risk of a severe adverse event after vaccination^a^, efficacy against COVID-19 infection^a^, efficacy against severe manifestation of COVID-19 infection^a^, and duration of protection after vaccination^a^	—	Out-of-pocket costs^a^	Incentives for completing vaccination^a^	Quarantine-free travel
George et al [64], 2022	7	Effectiveness^a^	Vaccination location^a^, waiting time at the vaccination site^a^, number of doses^a^, boosters required^a^, and vaccine origin^a^	—	Incentives for vaccination^a^	Effectiveness
Hazlewood et al [62], 2023	4	Effectiveness^a^, rare but serious risks^a^, and likelihood of having a flare^a^	Dosing^a^	—	—	Effectiveness
Hess et al [54], 2022	9	Estimated protection duration, risk of mild side effects, and risk of severe side effects	—	Fee	Exemption from international travel restrictions, risk of infection, and risk of serious illness, and population coverage	Effectiveness
Huang et al [48], 2021	4	Effectiveness^a^, risk of adverse reactions^a^, and duration of immunity^a^	—	—	Whether coworkers have been vaccinated^a^	Effectiveness
Igarashi et al [38], 2022	5	Safety^a^, efficacy^a^, and immunity duration^a^	—	Price^a^	Disease prevalence	Effectiveness
Krueger, and Daziano [58], 2022	9	Effectiveness^a^, protection period^a^, risk of severe side effects^a^, risk of mild side effects^a^, and incubation period^a^	Origin of the vaccine^a^, number of required doses^a^, and whether the vaccine has a booster against variants	Out-of-pocket cost^a^	—	Effectiveness
Leng et al [51], 2021	7	Vaccine effectiveness^a^, side effects^a^, and duration of vaccine protection^a^	Accessibility^a^, number of doses^a^, and vaccination sites^a^	—	Proportion of acquaintances vaccinated^a^	Effectiveness
Luyten et al [72], 2022	5	—	Age^a^, essential profession^a^, and medical risk group^a^	Cost to society^a^	Virus spreader^a^	Medical risk group
Li et al [74], 2021	6	Nonsevere adverse reactions^a^, efficacy^a^, and protection duration	Required number of doses^a^, and origin of the vaccine^a^	Out-of-pocket price^a^	—	Safety
Li et al [41], 2023	6	Adverse effect^a^, efficacy^a^, duration of vaccine effect^a^, and time for the vaccine to start working^a^	Vaccine varieties^a^	Cost of vaccination^a^	—	China: cost; The United States: effectiveness
Liu et al [31], 2021	6	Adverse effect^a^, efficacy^a^, duration of vaccine effect^a^, and time for the vaccine to start working	Vaccine varieties^a^	Cost of vaccination^a^	—	China: cost; the United States: effectiveness
McPhedran et al [73], 2022	4	—	Delivery mode^a^, appointment timing^a^, and proximity^a^	—	Sender^a^	SMS text message invitation sender
McPhedran et al [42], 2021	5	Level of protection offered^a^	Location in which the vaccine is administered^a^ and the number of doses needed for full protection^a^	—	Recommender of the vaccine^a^ and coverage in the media^a^	Effectiveness
Morillon and Poder [71], 2022	7	Effectiveness^a^, safety^a^, and duration^a^	Waiting time^a^, priority population^a^, and origin^a^	—	Recommendation^a^	Effectiveness
Mouter et al [43]	4	The percentage of vaccinated individuals protected against COVID-19^a^, the number of cases of mild side effects^a^, and the number of cases of severe side effects^a^	The month when the vaccine would become available to the respondent^a^	—	—	Safety
Mouter et al [44], 2022	6	Decrease in deaths, decrease in health damage, and decrease in households with income loss	Vaccination at home and vaccination when and where convenient	One-time tax increase	Vaccination ambassadors, pay €250 (US $280.75) if does not get vaccinated^a^, receive €100 (US $113) if gets vaccinated^a^, vaccination passport daily activities during outbreak^a,^ vaccination passport large events^a^, counseling if does not get vaccinated^a^, and mandatory testing at own cost if does not get vaccinated^a^	Mandatory testing at own cost if does not get vaccinated
Panchalingam and Shi [68], 2022	5	Risk of severe side effects^a^, and effectiveness^a^, and duration of vaccine-induced protection^a^	—	—	Risk of unvaccinated children requiring hospitalization for COVID-19^a^ and local coverage^a^	Safety
Prosser et al [49], 2023	6	Effectiveness^a^, mild common side effects^a^, and rare adverse events^a^	Number of doses^a^, total time required to get vaccinated^a^, and regulatory approval^a^	—	—	Effectiveness
Schwarzinger et al [34], 2021	4	Safety^a^ and efficacy^a^	Place to be vaccinated^a^ and country of vaccine manufacturer^a^	—	—	Region of vaccine manufacturer
Steinert et al [63], 2022	4	—	Age^a^	—	Employment status^a^, country of residence and health care system capacity^a^, and mortality risk^a^	Mortality risk
Teh et al [53], 2022	5	Effectiveness^a^ and risk of developing severe side effects^a^	Vaccination schedule during office hours^a^, distance from home to vaccination center^a^, and halal content^a^	—	—	Halal content
Tran et al [55]^c^, 2023	6	Immunity duration, effectiveness, and side effects	—	Cost of the vaccine	Limitations if not vaccinated and COVID-19 mortality rate	Mortality rate
Velardo et al [40], 2021	5	Efficacy^a^, risk of serious side effects per 100,000^a^, and duration of vaccine immunity^a^	Place of vaccine administration and location of vaccine manufacturer^a^	—	—	Effectiveness
Wang et al [61], 2022	6	Probability of fever, side effects^a^ and effectiveness^a^	Location of vaccination^a^, number of doses^a^, and origin of vaccine^a^	Price (CNY)^a^	—	Effectiveness
Wang et al [36], 2021	7	Probability of COVID-19 infection^a^ and probability of serious adverse event^a^	Brand^a^ and venue for vaccination^a^	—	Recommendations from professionals, quarantine for vaccinated travelers^a^, and vaccine uptake of people around^a^	Effectiveness
Wang et al [35] 2022	7	Efficacy^a^ and probability of serious adverse event^a^	Venue for vaccination and brand^a^	—	Recommendations from professionals, vaccination coverage among all children aged <18 years^a^, and vaccine uptake among acquaintances’ minor children	Effectiveness
Wang et al [52], 2022	6	Self-assessed vaccine-related side effects^a^, duration of vaccine protection^a^, and effectiveness^a^	Vaccination sites^a^	—	Risk perception^a^ and acquaintances vaccinated^a^	Safety
Wang et al [52], 2022	6	Effectiveness^a^, side effects^a^, and duration of protection^a^	Vaccination sites^a^	—	Perceived probability of infection of individuals or acquaintances^a^ and percentage of acquaintances vaccinated^a^	Effectiveness
Xiao et al [67], 2022	4	Effectiveness^a^, adverse reactions^a^, and protection period^a^	—	Price^a^	—	Effectiveness
Zhang et al [37], 2022	6	Efficacy^a^, duration^a^, adverse effect^a^, and time period when the vaccine starts working^a^	Varieties^a^	Cost^a^	—	Cost

^a^Attribute is significant (*P*<.05).

^b^Not available.

^c^The corresponding coefficients and *P* values are not provided.

### The Most Important Attribute Reported in DCE Studies

In total, 2 of the 5 multicountry studies did not report preferences for each country and were therefore excluded from the synthesis of the most important attribute. A total of 53 data points on COVID-19 vaccine preferences were collected from the study population of the corresponding country. In the *outcome* category, among the 30 attributes examined, effectiveness emerged as the most prominent, accounting for 40% (21/53) of the studies [31,35,36,38-42,48,50-52,57,58,60-62,64-67]. Safety was addressed in 13% (7/53) of the studies [33,43,47,56,59,68,69], while protection duration was mentioned in 4% (2/53) [11,50]. In the *process* category, 13 attributes were identified. Brand (1/53, 2%) [32], region of vaccine manufacturer (1/53, 2%) [34], and halal content (1/53, 2%) [53] were associated with vaccine production. In addition, waiting time for COVID-19 vaccination (1/53, 2%) [70] and vaccine frequency (1/53, 2%) [71] were considered. Furthermore, 3 (6%) studies on vaccine distribution prioritized vaccination for the medical risk group (1/53, 2%) [72], those who had a higher COVID-19 mortality risk (6/53, 11%) [63], and those who had the potential capacity to spread the virus (1/53, 2%) [72]. In the *cost* category, personal vaccination cost accounted for 6% (3/53) [31,37,41]. Among the *other* attributes (7/53, 13%), disease risk threat was of particular importance, including possible trends of the epidemic (1/53, 2%) [30] and COVID-19 mortality rate (1/53, 2%) [55]. In addition, incentives and penalties for vaccination were identified, including quarantine-free travel (1/53, 2%) [33] and mandatory testing at own expense if not vaccinated (1/53, 2%) [44]. Vaccine advice or support included vaccination invitation sender (1/53, 2%) [73] and recommenders (1/53, 2%) [46]. The proportion of friends and family members who had received the vaccine (1/53, 2%) [26] was also among the *other* attributes influencing decision-making (Table 2).

Although effectiveness remained the most important attribute, it is worth noting that variations in preferences were also observed among different subgroups. A higher proportion of studies conducted in LMICs (4/24, 17%) than in HICs (3/29, 10%) prioritized on safety (Multimedia Appendix 5). In addition, COVID-19 mortality risk was the second most important attribute (6/29, 21%) after effectiveness in HICs. Cost was considered to be another most important attribute (3/24, 13%) in LMICs. Interestingly, many other attributes also became more important as the pandemic progressed. Protection duration (2/24, 8%) emerged as one of the most important attributes during the pandemic wave. COVID-19 mortality risk (5/25, 20%) and cost (3/25, 12%) were considered as the most important attributes after the pandemic wave (Multimedia Appendix 6).

### Study Quality

The overall reporting quality was deemed acceptable but there is room for improvement. The PREFS scores of the 47 studies ranged from 2 to 4, with a mean of 3.23 (SD 0.52). No study scored 5. Most studies scored 3 (32/47, 68%) or 4 (13/47, 28%), while 2 studies (2/47, 4%) scored 2 (Multimedia Appendix 7 [11,26,30-74]).

## Discussion

### Principal Findings

This systematic review synthesizes existing data on preference for COVID-19 vaccine using DCE, with the aim of informing improvements in vaccine coverage and vaccine policy development. We identified 47 studies conducted in 29 countries, including 21 HICs and 8 LMICs. HICs had an adequate supply of vaccine since the early emergency availability of COVID-19 vaccine, and HICs had 1.5 times more doses of COVID-19 vaccinations than LMICs by September 2023 [85]. In total, 19 (40%) studies were conducted in China and 9 (19%) in the United States, demonstrating their significant contribution to the research and their leadership in vaccine research and development. Vaccine effectiveness and safety were the most important attributes in DCEs, although preferences differed among subgroups.

Recent years have seen new trends in the design, implementation, and validation of the DCE. For example, most studies (40/47, 85%) reported that the DCE was administered through web-based surveys, which have become a quick and cost-effective way to collect DCE data [66]. Almost half of the studies (25/47, 53%) did not report a pilot test. However, piloting in multiple stages throughout the development of a DCE is conducive to identifying appropriate and understandable attributes, considering whether participants can effectively evaluate the full profiles, and producing an efficient design [21,86,87].

Overall, vaccine effectiveness and safety have emerged as the most commonly investigated attributes in the *outcome* category. Despite heterogeneity in preferences across subpopulations, effectiveness remains the primary driver for COVID-19 vaccination across the studies [31,35,36,38-42,48,50,51,57,58,60-62,64-67], similar to the previous findings [18]. A study conducted in India and Europe found that respondents’ preference for the COVID-19 vaccine increased with effectiveness and peaked at 95% effectiveness [45]. Another study conducted among university staff and students in South Africa found that vaccine effectiveness not only was a concern but also significantly influenced vaccine choice behavior [64]. Interestingly, a nationwide stated choice survey in the United States found a strong interaction between effectiveness and other attributes [58]. These findings support the ongoing efforts to maximize vaccine effectiveness while emphasizing the importance of communicating information on vaccine effectiveness to the target population for promotion [62].

Safety has also been identified as a crucial factor influencing the acceptance of COVID-19 vaccine [33,43,47,56,59,68,69]. One study indicated that the likelihood of the general public choosing vaccines with low or moderate side effects increased by 75% and 63%, respectively, compared with vaccines with high side effects. While the likelihood changed within a 30% range when most attributes other than effectiveness and safety were changed [69]. In addition, respondents in Australia expressed a willingness to wait an additional 0.04 and 1.2 months to reduce the incidence of mild and severe adverse events by 1/10,000, respectively [56].

Similar to the results of previous systematic reviews of DCEs for various vaccines [18,19], the most common predictors of COVID-19 vaccine acceptance are effectiveness and safety, particularly during the rapid development and rollout of COVID-19 vaccines, which essentially boils down to trust in the vaccine [31]. Respondents expressed the importance of having a safe and effective COVID-19 vaccine available as soon as possible, but the majority preferred to wait a few months to observe the experience of others rather than be the first in line [43]. Therefore, collaborating to enhance vaccine effectiveness while reducing the risk of severe side effects could be a highly effective strategy to address vaccine hesitancy and augment vaccine desirability. Dissemination of this important vaccine-related information by governments and health care institutions, along with effective communication by health care professionals, can help build public trust and ultimately increase vaccination rates [69]. However, these inherent vaccine attributes are typically beyond the control of a vaccination program, and given the ongoing mutations of SARS-CoV-2, it is challenging to predict the effectiveness of the vaccines currently in development [66]. Global collaboration between scientists and pharmaceutical companies is therefore essential to improve vaccine effectiveness and minimize side effects [41].

Vaccine production, including its origin, brand, vaccine frequency, and content, are key considerations in the *process* category. Vaccine brand also has a significant impact on vaccine choice [32], independent of effectiveness and safety, due to factors such as reputation, country of origin, technological advances, and reported side effects associated with the brands [35]. For vaccine origin, some studies found that participants preferred domestic vaccines to imported vaccines, which may depend on the availability or the approval of vaccines in different countries [31,41,50] or the incidence of side effects among different types of COVID-19 vaccines [37]. However, some studies found that imported vaccines were more likely to be accepted than domestically produced vaccines, which may be attributed to less trust in domestically produced vaccines [57,66]. A study on vaccine preferences among the Malaysian population found that the composition and production process of the COVID-19 vaccine, which complied with Islamic dietary requirements (ie, halal content) was an important factor for many Malaysians when deciding whether to be vaccinated. This underscores the substantial influence of religion on vaccine choice [53].

Vaccine frequency was emphasized to play an important role in the choice of COVID-19 vaccine among the US public, while the 90% efficacy with low side effect rate of the COVID-19 vaccine was set. The prospect of vaccinating once to get lifelong immunity was very attractive, reflecting the fact that people were effort minimizers [71]. This is similar to the nature of the 2 studies referenced in the outcome attribute, where the protection duration is prioritized. Given the threat of COVID-19, people expect the protection duration to be as long as possible [11,50].

When vaccine supply is limited, people tend to prioritize vaccination for those who are more susceptible to the disease, have higher mortality rates from infectious diseases, or have greater potential to spread the virus. A study in Iran found that individuals tend to prioritize vaccination for those in the community with higher potential for virus transmission [57]. In addition, results from a study in 6 European countries revealed unanimous agreement among respondents that candidates with higher mortality and infection risks should be prioritized for vaccination [63]. While another study conducted among Belgians also found that respondents would prioritize populations at higher medical risk [72].

Cost was another important factor influencing COVID-19 vaccine preferences, mostly related to out-of-pocket costs [31,37,41]. In 2 studies comparing public preferences for COVID-19 vaccines in China and the United States, vaccine efficacy emerged as the most important driver for the American public, whereas the cost of vaccination had the greatest impact on the Chinese public. This difference was likely due to the relatively stable pandemic situation in China at the time and the lower perceived risk of COVID-19. As a result, the Chinese population was more price sensitive and reluctant to pay for vaccination [31,37,41].

For the *other* category, several different attributes were highlighted, depending on the specific population or situation. When people perceive the threat of a disease, their desire to be vaccinated becomes more urgent. In a study among health care workers in China, participants’ expectations about the future development of COVID-19 had a greater impact on their decision to be vaccinated than their perceived risk of infection or actual case rates, which may have been influenced by their previous experience with seasonal influenza vaccination [30]. The mortality rate of COVID-19 was considered the most influential factor in the uptake of COVID-19 booster shots in Vietnam. This study was conducted during a pandemic wave in Vietnam, which may have led to an increased perception of public health risks and a greater inclination toward COVID-19 vaccination [55]. To achieve herd immunity, government authorities can implement policies of incentives and penalties for vaccination to encourage population-wide uptake. A study conducted in the Netherlands revealed that respondents particularly disliked policies that penalized those who were not vaccinated, such as mandatory testing at their own expense if they were not vaccinated [44]. Instead, they favored policies that rewarded vaccination, such as giving vaccinated individuals additional privileges through a vaccination passport. This finding is consistent with a study in Hong Kong, which found that quarantine-free travel was considered the most important motivator among university students and staff, given their frequent engagement in international travel [33].

The source of vaccine information also influences vaccine decision-making [30]. Variation in the sender of vaccination appointment invitation via SMS text messaging and recommenders may potentially influence the public’s willingness to vaccinate against a disease [30,46,73]. Furthermore, the acceptance of vaccines was observed to change as the firsthand information about vaccine side effects and effectiveness was provided by friends and family in India [26].

In HICs, COVID-19 mortality risk was the second most important attribute after effectiveness, as respondents in all 6 high-income European countries from a study of public preferences for COVID-19 vaccine distribution prioritized candidates with higher mortality risks [63]. However, individuals from LMICs appeared to be more concerned about vaccine safety than those from HICs. This may be related to greater confidence in vaccine safety in HICs due to the earlier initiation and higher rates of COVID-19 vaccination [85]. In contrast, in some LMICs, vaccine safety was reported as the main reason influencing the willingness to vaccinate due to the rapid development of the COVID-19 vaccines [26,43,47,59,68,69,74,88].

Interestingly, the preference for COVID-19 vaccines may also have changed as the pandemic progressed [63]. Similarly, effectiveness remained the most important attribute in all periods, possibly due to the continuing severity of the pandemic and the fear of the possible emergence of new coronavirus strains [43]. Before the pandemic wave, the information on vaccine effectiveness was limited [26], but people still considered vaccine effectiveness to be the most important driver of vaccination. However, during the pandemic, the public’s perception of the health risk increased. As vaccines were introduced and used, people seemed to become more concerned about the duration of vaccine protection and preferred a longer vaccine protection [11,50]. After the pandemic wave, as the pandemic situation gradually stabilized, cost, combined with their perception of the risk of susceptibility, became more important in their preferences. However, despite this shift, most of the public still believed that people who are at higher risk of infection or death should be vaccinated first [63].

### Limitations

Our study had several limitations. First, not all studies used the same attributes and levels, which limited our ability to perform a quantitative synthesis and directly compare the estimates of model parameters. Instead, we qualitatively synthesized and summarized the range of attributes that may be useful in the formative stage of attribute selection in future DCE surveys investigating the preference for COVID-19 vaccine. Second, although DCEs have been shown to be a valid method for eliciting preferences, the experiment may not represent real market choices but rather hypothetical scenarios with plausible and realistic attributes. However, it offers opportunities to evaluate vaccines that are not yet available in the market or to specific population [68]. Third, the commonly used classification of outcome, cost, and process was used in order to better explain the public’s preference for vaccine attributes. However, several attributes could not be properly classified, and a fourth category (ie, *other* attributes) had to be added [19]. Meanwhile, the variety of attributes included may make it difficult to appropriately name and interpret this category as a whole. Fifth, the PREFS checklist is limited to 5 questions and fails to elicit several criteria that should be reported in DCE studies. Also, it does not provide sufficient tools to assess the biases in a DCE, such as selection bias and nonresponse bias [79,89]. Finally, although there was no specific theoretical framework to structure our qualitative analysis from the 4 identified categories, our classification was based on previous studies [18,19,82,90,91] and our own findings. This synthesis led us to categorize attributes into 4 main classes, providing a clear structure for analyzing and presenting participants’ vaccine preferences and making it easier to compare their preferences across different studies.

### Conclusions

In conclusion, this systematic review synthesized the global evidence on preferences for COVID-19 vaccines using the DCE methodology. Vaccine effectiveness and safety were found to be the main drivers for COVID-19 vaccination, highlighting the importance of global collaboration to improve vaccine effectiveness and minimize side effects, as well as the importance of communicating this vaccine-related information to the public to maximize the uptake of COVID-19 vaccines. The subgroup analyses emphasized the importance of differences in vaccine preference of specific populations and time periods in optimizing the acceptance of COVID-19 vaccines. These findings may serve as valuable insights for government agencies involved in the social mobilization process for COVID-19 vaccination. However, the response to the pandemic is a continuous learning process [92]. It is crucial for policy makers to consider preference evidence when designing policies to promote vaccination.

## Appendix

Multimedia Appendix 1 PRISMA (Preferred Reporting Items for Systematic Reviews and Meta-Analyses) 2020 checklist.

Multimedia Appendix 2 Search strategies.

Multimedia Appendix 3 Attributes included in each category.

Multimedia Appendix 4 The detailed distribution of the study period across countries.

Multimedia Appendix 5 Preference for COVID-19 vaccines among high-income countries and low- and middle-income countries (n=53).

Multimedia Appendix 6 Preference for COVID-19 vaccines in the different study periods (n=53).

Multimedia Appendix 7 Assessment of 47 included studies quality using the Purpose, Respondents, Explanation, Findings, and Significance checklist.

## Abbreviations

DCE: discrete choice experiment
HIC: high-income country
LMIC: low- and middle-income country
PREFS: Purpose, Respondents, Explanation, Findings, and Significance
PRISMA: Preferred Reporting Items for Systematic Reviews and Meta-Analyses

## Data Availability

All data relevant to the study are included in the article or uploaded as supplemental information. Data sets of this study are available upon reasonable request to the corresponding author.
