# Supplementary material for: Preferences for COVID-19 Vaccines: Systematic Literature Review of Discrete Choice Experiments
Source: JMIR Public Health Surveill. 2024 Jul 29;10:e56546. doi: 10.2196/56546 (PMC11319885; doi:10.2196/56546)
Supplement: Multimedia Appendix 2 [file publichealth_v10i1e56546_app2.docx]

**Multimedia Appendix 2. Search strategies**

**1.1 Pubmed**

("discrete choice experiment*"[Text Word] OR "discrete choice analysis"[Text Word] OR "discrete choice model*"[Text Word] OR "choice experiment*"[Text Word] OR "choice modelling"[Text Word] OR "choice modeling"[Text Word] OR "DCE"[Text Word] OR "conjoint choice experiment*"[Text Word] OR "choice-based conjoint analysis"[Text Word] OR "choice based conjoint analysis"[Text Word] OR "conjoint analys*"[Text Word] OR conjoint[Text Word] OR "stated choice"[Text Word] OR "stated-choice"[Text Word] OR "stated preference"[Text Word] OR "stated-preference"[Text Word] OR "stated-preference method"[Text Word] OR "stated preference method"[Text Word] OR "preference-based method"[Text Word] OR "preference-based approach"[Text Word] OR "preference elicitation"[Text Word] OR "best-worst"[Text Word] OR "worst-best"[Text Word] OR "best-worst scaling"[Text Word] OR "best worst scaling"[Text Word] OR "worstbest scale"[Text Word] OR "worst best scaling"[Text Word] OR "best-worst scale"[Text Word] OR "best worst scale"[Text Word] OR "worst best scale"[Text Word] OR "maximum difference scaling"[Text Word] OR "maxdiff scaling"[Text Word] OR "maximum difference"[Text Word] OR Maxdiff[Text Word] OR "max diff"[Text Word] OR "paired comparison*"[Text Word] OR "pairwise choice*"[Text Word] OR "part-worth utilities"[Text Word] OR ("patient preference"[MeSH Terms] OR "patient preference"[Text Word]) OR "patient weighting"[Text Word] OR "patient rating"[Text Word] OR "patient ranking"[Text Word] OR "patient perspective"[Text Word] OR "patient priorities"[Text Word] OR "functional measurement"[Text Word] OR "direct preference"[Text Word] OR "direct assessment"[Text Word]) AND ("covid-19"[MeSH Terms] OR "SARS-CoV-2"[MeSH Terms] OR "covid- 19"[Text Word] OR "covid-19"[Text Word] OR "COVID19"[Text Word] OR "COVID 19"[Text Word] OR "COVID-2019"[Text Word] OR "2019-nCoV"[Text Word] OR "2019 nCoV"[Text Word] OR "COVID2019"[Text Word] OR "covid 2019"[Text Word] OR "covid 2019"[Text Word] OR "novel coronavirus"[Text Word] OR "new coronavirus"[Text Word] OR "novel corona virus"[Text Word] OR "new corona virus"[Text Word] OR "SARS-CoV-2"[Text Word] OR "SARSCoV2"[Text Word] OR "SARS Coronavirus 2"[Text Word]OR "SARS-CoV2"[Text Word] OR "2019nCoV"[Text Word] OR "2019- nCoV"[Text Word] OR "2019 coronavirus"[Text Word] OR "2019 corona virus"[Text Word] OR "coronavirus disease 2019"[Text Word] OR "coronavirus disease-19"[Text Word] OR "severe acute respiratory syndrome coronavirus 2"[Multimedia Appendixary Concept] OR "severe acute respiratory syndrome coronavirus 2"[Text Word] OR "sars-coronavirus-2"[Text Word] OR "SARS Coronavirus 2"[Text Word] OR "corona virus disease 2019"[Text Word] OR "coronavirus disease 19"[Text Word]) AND ("COVID-19 Vaccines"[MeSH Terms] OR "vaccin*"[Text Word] OR inocul*[Text Word])

**1.2 Embase**

#1 'covid 19'/exp OR 'covid 19' OR 'covid 19':ti,ab,kw OR 'covid 2019'/exp OR 'covid 2019' OR 'covid 2019':ti,ab,kw OR 'coronavirus disease 2019'/exp OR 'coronavirus disease 2019' OR 'coronavirus disease 2019':ti,ab,kw OR 'novel coronavirus':ti,ab,kw OR 'new coronavirus':ti,ab,kw OR 'novel corona virus':ti,ab,kw OR 'new corona virus':ti,ab,kw OR 'sars-cov-2'/exp OR 'sars-cov-2' OR 'sars-cov-2':ti,ab,kw OR 'severe acute respiratory syndrome coronavirus 2'/exp OR 'severe acute respiratory syndrome coronavirus 2' OR 'severe acute respiratory syndrome coronavirus 2':ti,ab,kw OR 'sars-coronavirus-2'/exp OR 'sars-coronavirus-2' OR 'sars-coronavirus-2':ti,ab,kw OR '2019ncov':ti,ab,kw OR '2019 ncov':ti,ab,kw OR '2019-ncov'/exp OR '2019-ncov' OR '2019-ncov':ti,ab,kw OR '2019 coronavirus':ti,ab,kw OR '2019 corona virus':ti,ab,kw OR 'corona virus disease 2019':ti,ab,kw OR 'coronavirus disease 19':ti,ab,kw OR 'coronavirus disease-19':ti,ab,kw OR 'covid19':ti,ab,kw OR 'covid-2019':ti,ab,kw OR 'covid2019':ti,ab,kw OR 'sarscov2':ti,ab,kw OR 'sars-cov2':ti,ab,kw OR 'SARS Coronavirus 2':ti,ab,kw OR '2019- ncov':ti,ab,kw

#2 'vaccines'/exp OR 'vaccine'/exp OR 'vaccination'/exp OR vaccin*:ti,ab,kw OR 'inoculation'/exp OR inocul*:ti,ab,kw

#3 'discrete choice experiment*':ti,ab,kw OR 'discrete choice analysis':ti,ab,kw OR 'discrete choice model*':ti,ab,kw OR 'choice experiment*':ti,ab,kw OR 'choice modelling':ti,ab,kw OR 'choice modeling':ti,ab,kw OR 'dce' OR 'conjoint choice experiment*':ti,ab,kw OR 'choice-based conjoint analysis':ti,ab,kw OR 'choice based conjoint analysis':ti,ab,kw OR 'conjoint analys*':ti,ab,kw OR 'conjoint':ti,ab,kw OR 'stated choice':ti,ab,kw OR 'stated-choice':ti,ab,kw OR 'stated preference':ti,ab,kw OR 'stated-preference':ti,ab,kw OR 'stated-preference method':ti,ab,kw OR 'stated preference method':ti,ab,kw OR 'preference-based method':ti,ab,kw OR 'preference-based approach':ti,ab,kw OR 'preference elicitation':ti,ab,kw OR 'best-worst':ti,ab,kw OR 'worst-best':ti,ab,kw OR 'best-worst scaling':ti,ab,kw OR 'best worst scaling'/exp OR 'best worst scaling':ti,ab,kw OR 'worstbest scale':ti,ab,kw OR 'worst best scaling':ti,ab,kw OR 'best-worst scale':ti,ab,kw OR 'best worst scale':ti,ab,kw OR 'worst best scale':ti,ab,kw OR 'maximum difference scaling':ti,ab,kw OR 'maxdiff scaling':ti,ab,kw OR 'maximum difference':ti,ab,kw OR 'maxdiff':ti,ab,kw OR 'max diff':ti,ab,kw OR 'paired comparison*':ti,ab,kw OR 'pairwise choice*':ti,ab,kw OR 'part-worth utilities':ti,ab,kw OR 'patient preference'/exp OR 'patient preference':ti,ab,kw OR 'patient weighting':ti,ab,kw OR 'patient rating':ti,ab,kw OR 'patient ranking':ti,ab,kw OR 'patient perspective'/exp OR 'patient perspective':ti,ab,kw OR 'patient priorities':ti,ab,kw OR 'functional measurement':ti,ab,kw OR 'direct preference':ti,ab,kw OR 'direct assessment':ti,ab,kw

**1.3 Web of Science**

TS=("discrete choice experiment*" OR "discrete choice analysis" OR "discrete choice model*" OR "choice experiment*" OR "choice modelling" OR "choice modeling" OR "DCE" OR "conjoint choice experiment*" OR "choice-based conjoint analysis" OR "choice based conjoint analysis" OR "conjoint analys*" OR conjoint OR "stated choice" OR "stated-choice" OR "stated preference" OR "stated-preference" OR "stated-preference method" OR "stated preference method" OR "preference-based method" OR "preference-based approach" OR "preference elicitation" OR "best-worst" OR "worst-best" OR "best-worst scaling" OR "best worst scaling" OR "worstbest scale" OR "worst best scaling" OR "best-worst scale" OR "best worst scale" OR "worst best scale" OR "maximum difference scaling" OR " maxdiff scaling" OR "maximum difference" OR maxdiff OR "max diff" OR "paired comparison*" OR "pairwise choice*" OR "part-worth utilities" OR "patient preference" OR "patient weighting" OR "patient rating" OR "patient ranking" OR "patient perspective" OR "patient priorities" OR "functional measurement" OR "direct preference" OR "direct assessment") AND TS=("covid-19" OR "covid- 19" OR "COVID19" OR "COVID 19" OR "COVID-2019" OR "2019-nCoV" OR "covid019" OR "covid 2019" OR "covid 2019" OR "novel coronavirus" OR "new coronavirus" OR "novel corona virus" OR "new corona virus" OR "SARS-CoV-2" OR "SARSCoV2" OR "SARS-CoV2" OR "SARS Coronavirus 2" OR "2019ncovr" OR "2019- nCoV" OR "2019 nCoV" OR "2019 coronavirus" OR "2019 corona virus" OR "coronavirus disease 2019" OR "coronavirus disease 19" OR "coronavirus disease-19" OR "severe acute respiratory syndrome coronavirus 2" OR "sars-coronavirus-2" OR "corona virus disease 2019") AND TS=("vaccin*" OR "inocul*")

**1.4 Scopus**

( TITLE-ABS-KEY ( "discrete choice experiment*" OR "discrete choice analysis" OR "discrete choice model*" OR "choice experiment*" OR "choice modelling" OR "choice modeling" OR "DCE" OR "conjoint choice experiment*" OR "choice-based conjoint analysis" OR "choice based conjoint analysis" OR "conjoint analys*" OR conjoint OR "stated choice" OR "stated-choice" OR "stated preference" OR "stated-preference" OR "stated-preference method" OR "stated preference method" OR "preference-based method" OR "preference-based approach" OR "preference elicitation" OR "best-worst" OR "worst-best" OR "best-worst scaling" OR "best worst scaling" OR "worstbest scale" OR "worst best scaling" OR "best-worst scale" OR "best worst scale" OR "worst best scale" OR "maximum difference scaling" OR " maxdiff scaling" OR "maximum difference" OR maxdiff OR "max diff" OR "paired comparison*" OR "pairwise choice*" OR "part-worth utilities" OR "patient preference" OR "patient weighting" OR "patient rating" OR "patient ranking" OR "patient perspective" OR "patient priorities" OR "functional measurement" OR "direct preference" OR "direct assessment") AND TITLE-ABS-KEY ( "covid-19" OR "covid- 19" OR "COVID19" OR "COVID 19" OR "COVID-2019" OR "2019-nCoV" OR "covid019" OR "covid 2019" OR "covid 2019" OR "novel coronavirus" OR "new coronavirus" OR "novel corona virus" OR "new corona virus" OR "SARS-CoV-2" OR "SARSCoV2" OR "SARS-CoV2" OR "SARS Coronavirus 2" OR "2019ncovr" OR "2019 ncov" OR "2019- nCoV" OR "2019 coronavirus" OR "2019 corona virus" OR "coronavirus disease 2019" OR "coronavirus disease 19" OR "coronavirus disease-19" OR "severe acute respiratory syndrome coronavirus 2" OR "sars-coronavirus-2" OR "corona virus disease 2019" ) AND TITLE-ABS-KEY ( "vaccin*" OR "inocul*" ) )

**1.5 CINAHL Plus**

TX ( "discrete choice experiment*" OR "discrete choice analysis" OR "discrete choice model*" OR "choice experiment*" OR "choice modelling" OR "choice modeling" OR "DCE" OR "conjoint choice experiment*" OR "choice-based conjoint analysis" OR "choice based conjoint analysis" OR "conjoint analys*" OR conjoint OR "stated choice" OR "stated-choice" OR "stated preference" OR "stated-preference" OR "stated-preference method" OR "stated preference method" OR "preference-based method" OR "preference-based approach" OR "preference elicitation" OR "best-worst" OR "worst-best" OR "best-worst scaling" OR "best worst scaling" OR "worstbest scale" OR "worst best scaling" OR "best-worst scale" OR "best worst scale" OR "worst best scale" OR "maximum difference scaling" OR " maxdiff scaling" OR "maximum difference" OR maxdiff OR "max diff" OR "paired comparison*" OR "pairwise choice*" OR "part-worth utilities" OR "patient preference" OR "patient weighting" OR "patient rating" OR "patient ranking" OR "patient perspective" OR "patient priorities" OR "functional measurement" OR "direct preference" OR "direct assessment") AND TX ( "covid-19" OR "covid- 19" OR "COVID19" OR "COVID 19" OR "COVID-2019" OR "2019-nCoV" OR "covid019" OR "covid 2019" OR "covid 2019" OR "novel coronavirus" OR "new coronavirus" OR "novel corona virus" OR "new corona virus" OR "SARS-CoV-2" OR "SARSCoV2" OR "SARS-CoV2" OR "SARS Coronavirus 2" OR "2019ncovr" OR "2019- nCoV" OR "2019 ncov" OR "2019 coronavirus" OR "2019 corona virus" OR "coronavirus disease 2019" OR "coronavirus disease 19" OR "coronavirus disease-19" OR "severe acute respiratory syndrome coronavirus 2" OR "sars-coronavirus-2" OR "corona virus disease 2019" ) AND TX ( "vaccin*" OR "inocul*" )
