# Supplementary material for: Preferences for COVID-19 Vaccines: Systematic Literature Review of Discrete Choice Experiments
Source: JMIR Public Health Surveill. 2024 Jul 29;10:e56546. doi: 10.2196/56546 (PMC11319885; doi:10.2196/56546)
Supplement: Multimedia Appendix 3 [file publichealth_v10i1e56546_app3.docx]

**Multimedia Appendix 3. Attributes included in each category**

| Author, year | Key categories | Subcategories | The article's expression | Title |
| --- | --- | --- | --- | --- |
| Asim et al., 2023 | Outcome | Vaccine safety | safety^a^ | COVID-19 Vaccination Preferences Among Non-Chinese Migrants in Hong Kong: Discrete Choice Experiment |
| Bansal et al., 2022 | Outcome | Vaccine safety | Side effects^a^ | COVID-19 vaccine preferences in India |
| Blaga et al., 2023 | Outcome | Vaccine safety | the type of possible side effect^a^ | Examination of Preferences for COVID-19 Vaccines in Hungary Based on Their Properties-Examining the Impact of Pandemic Awareness with a Hybrid Choice Approach |
| Chen et al., 2023 | Outcome | Vaccine safety | the risk of adverse effects^a^ | The COVID-19 vaccination decision-making preferences of elderly people: a discrete choice experiment |
| Chen et al., 2021 | Outcome | Vaccine safety | adverse effect^a^ | Public preference and vaccination willingness for COVID-19 vaccine in China |
| Craig, 2021 | Outcome | Vaccine safety | Risk of severe side effects^a^ | United States COVID-19 Vaccination Preferences (CVP): 2020 Hindsight |
| Darrudi et al., 2022 | Outcome | Vaccine safety | risk of severe complications^a^ | Public Preferences and Willingness to Pay for a COVID-19 Vaccine in Iran: A Discrete Choice Experiment |
| Daziano, 2022 | Outcome | Vaccine safety | Number of people out of 10 with mild side effects^a^ | A choice experiment assessment of stated early response to COVID-19 vaccines in the USA |
| Daziano, 2022 | Outcome | Vaccine safety | Number of people out of 1000000 with severe side effects^a^ | A choice experiment assessment of stated early response to COVID-19 vaccines in the USA |
| Díaz Luévano et al., 2021 | Outcome | Vaccine safety | safety^a^ | Quantifying healthcare and welfare sector workers' preferences around COVID-19 vaccination: a cross-sectional, single-profile discrete-choice experiment in France |
| Dong et al., 2020 | Outcome | Vaccine safety | Adverse event^a^ | Public preference for COVID-19 vaccines in China: A discrete choice experiment |
| Dong et al., 2022 | Outcome | Vaccine safety | adverse effects^a^ | Acceptance of and Preference for COVID-19 Vaccination in India, the United Kingdom, Germany, Italy, and Spain: An International Cross-Sectional Study |
| Donin et al., 2022 | Outcome | Vaccine safety | Risk of mild side effects^a^ | Factors Affecting Young Adults' Decision Making to Undergo COVID-19 Vaccination: A Patient Preference Study |
| Fu et al., 2020 | Outcome | Vaccine safety | Vaccine safety^a^ | Acceptance of and preference for COVID-19 vaccination in healthcare workers: a comparative analysis and discrete choice experiment |
| Fung et al., 2022 | Outcome | Vaccine safety | risk of a mild or moderate adverse event after vaccination^a^ | COVID-19 Vaccination Preferences of University Students and Staff in Hong Kong |
| Fung et al., 2022 | Outcome | Vaccine safety | risk of a severe adverse event after vaccination^a^ | COVID-19 Vaccination Preferences of University Students and Staff in Hong Kong |
| Hazlewood et al., 2023 | Outcome | Vaccine safety | Rare but serious risks^a^ | Preferences for COVID-19 Vaccination in People With Chronic Immune-Mediated Inflammatory Diseases |
| Hazlewood et al., 2023 | Outcome | Vaccine safety | Likelihood of having a flare^a^ | Preferences for COVID-19 Vaccination in People With Chronic Immune-Mediated Inflammatory Diseases |
| Huang et al., 2021 | Outcome | Vaccine safety | risk of adverse reactions (5% vs 10%)^a^ | COVID-19 vaccine coverage, concerns, and preferences among Chinese ICU clinicians: a nationwide online survey |
| Krueger et al., 2022 | Outcome | Vaccine safety | risk of severe side effects^a^ | Stated choice analysis of preferences for COVID-19 vaccines using the Choquet integral |
| Krueger et al., 2022 | Outcome | Vaccine safety | risk of mild side effects^a^ | Stated choice analysis of preferences for COVID-19 vaccines using the Choquet integral |
| Leng et al., 2021 | Outcome | Vaccine safety | side-effects^a^ | Individual preferences for COVID-19 vaccination in China |
| Li et al., 2021 | Outcome | Vaccine safety | non-severe adverse reactions^a^ | COVID-19 vaccine preferences among university students in Hong Kong: a discrete choice experiment |
| Li et al., 2023 | Outcome | Vaccine safety | Adverse effect^a^ | Understanding influencing attributes of COVID-19 vaccine preference and willingness-to-pay among Chinese and American middle-aged and elderly adults: A discrete choice experiment and propensity score matching study |
| Liu et al., 2021 | Outcome | Vaccine safety | Adverse effect^a^ | A Comparison of Vaccine Hesitancy of COVID-19 Vaccination in China and the United States |
| Morillon et al., 2022 | Outcome | Vaccine safety | safety^a^ | Public Preferences for a COVID-19 Vaccination Program in Quebec: A Discrete Choice Experiment |
| Mouter et al., 2022 | Outcome | Vaccine safety | The number of cases of mild side effects^a^ | "Please, you go first!" preferences for a COVID-19 vaccine among adults in the Netherlands |
| Mouter et al., 2022 | Outcome | Vaccine safety | The number of cases of severe side effects^a^ | "Please, you go first!" preferences for a COVID-19 vaccine among adults in the Netherlands |
| Panchalingam et al., 2022 | Outcome | Vaccine safety | risk of severe side effects^a^ | Parental refusal and hesitancy of vaccinating children against COVID-19: Findings from a nationally representative sample of parents in the U.S |
| Prosser et al., 2023 | Outcome | Vaccine safety | mild common side effects^a^ | A Discrete Choice Analysis Comparing COVID-19 Vaccination Decisions for Children and Adults |
| Prosser et al., 2023 | Outcome | Vaccine safety | Rare adverse events^a^ | A Discrete Choice Analysis Comparing COVID-19 Vaccination Decisions for Children and Adults |
| Schwarzinger et al., 2021 | Outcome | Vaccine safety | vaccine safety^a^ | COVID-19 vaccine hesitancy in a representative working-age population in France: a survey experiment based on vaccine characteristics |
| Teh et al., 2022 | Outcome | Vaccine safety | the risk of developing severe side effects^a^ | Malaysian public preferences and decision making for COVID-19 vaccination: A discrete choice experiment |
| Tran et al., 2023 | Outcome | Vaccine safety | side effects^a^ | Preference and Willingness to Pay for the Regular COVID-19 Booster Shot in the Vietnamese Population: Theory-Driven Discrete Choice Experiment |
| Wang et al., 2021 | Outcome | Vaccine safety | Probability of serious adverse event^a^ | Influence of Vaccination Characteristics on COVID-19 Vaccine Acceptance Among Working-Age People in Hong Kong, China: A Discrete Choice Experiment |
| Wang et al., 2022 | Outcome | Vaccine safety | Probability of serious adverse event^a^ | Impact of information framing and vaccination characteristics on parental COVID-19 vaccine acceptance for children: a discrete choice experiment |
| Wang et al., 2022 | Outcome | Vaccine safety | Self-assessed vaccine-related side effects^a^ | Student COVID-19 vaccination preferences in China: A discrete choice experiment |
| Wang et al., 2022 | Outcome | Vaccine safety | vaccine-related side effects^a^ | Individual Preferences for COVID-19 Vaccination under the China's 2021 National Vaccination Policy: A Discrete Choice Experiment Study |
| Xiao et al., 2022 | Outcome | Vaccine safety | adverse reactions^a^ | Attribute nonattendance in COVID-19 vaccine choice: A discrete choice experiment based on Chinese public preference |
| Zhang et al., 2022 | Outcome | Vaccine safety | Adverse effect^a^ | Personality Effects on Chinese Public Preference for the COVID-19 Vaccination: Discrete Choice Experiment and Latent Profile Analysis Study |
| Borriello et al., 2021 | Outcome | Vaccine safety | Mild side effects^a^ | Location, location, location: a discrete choice experiment to inform COVID-19 vaccination programme delivery in the UK |
| Borriello et al., 2021 | Outcome | Vaccine safety | Major side effects^a^ | Location, location, location: a discrete choice experiment to inform COVID-19 vaccination programme delivery in the UK |
| Hess et al., 2022 | Outcome | Vaccine safety | Risk of mild side effects | Vaccination or NPI? A conjoint analysis of German citizens' preferences in the context of the COVID-19 pandemic |
| Hess et al., 2022 | Outcome | Vaccine safety | Risk of severe side effects | Vaccination or NPI? A conjoint analysis of German citizens' preferences in the context of the COVID-19 pandemic |
| Igarashi et al., 2022 | Outcome | Vaccine safety | safety^a^ | Vaccination or NPI? A conjoint analysis of German citizens' preferences in the context of the COVID-19 pandemic |
| Velardo et al., 2021 | Outcome | Vaccine safety | Risk of serious side effects per 100000^a^ | How should COVID-19 vaccines be distributed between the Global North and South: a discrete choice experiment in six European countries |
| Wang et al., 2022 | Outcome | Vaccine safety | Probability of fever side-effects (%)^a^ | How should COVID-19 vaccines be distributed between the Global North and South: a discrete choice experiment in six European countries |
| Asim et al., 2023 | Outcome | Vaccine effectiveness | efficacy^a^ | COVID-19 Vaccination Preferences Among Non-Chinese Migrants in Hong Kong: Discrete Choice Experiment |
| Bansal et al., 2022 | Outcome | Vaccine effectiveness | Effectiveness of vaccine^a^ | COVID-19 vaccine preferences in India |
| Blaga et al., 2023 | Outcome | Vaccine effectiveness | the effectiveness of the vaccine^a^ | Examination of Preferences for COVID-19 Vaccines in Hungary Based on Their Properties-Examining the Impact of Pandemic Awareness with a Hybrid Choice Approach |
| Chen et al., 2023 | Outcome | Vaccine effectiveness | effectiveness^a^ | The COVID-19 vaccination decision-making preferences of elderly people: a discrete choice experiment |
| Chen et al., 2021 | Outcome | Vaccine effectiveness | protection rate^a^ | Public preference and vaccination willingness for COVID-19 vaccine in China |
| Craig, 2021 | Outcome | Vaccine effectiveness | Vaccine effectiveness^a^ | United States COVID-19 Vaccination Preferences (CVP): 2020 Hindsight |
| Darrudi et al., 2022 | Outcome | Vaccine effectiveness | effectiveness^a^ | Public Preferences and Willingness to Pay for a COVID-19 Vaccine in Iran: A Discrete Choice Experiment |
| Daziano, 2022 | Outcome | Vaccine effectiveness | Effectiveness^a^ | A choice experiment assessment of stated early response to COVID-19 vaccines in the USA |
| Díaz Luévano et al., 2021 | Outcome | Vaccine effectiveness | efficacy^a^ | Quantifying healthcare and welfare sector workers' preferences around COVID-19 vaccination: a cross-sectional, single-profile discrete-choice experiment in France |
| Dong et al., 2020 | Outcome | Vaccine effectiveness | Effectiveness (%)^a^ | Public preference for COVID-19 vaccines in China: A discrete choice experiment |
| Dong et al., 2022 | Outcome | Vaccine effectiveness | effificacy^a^ | Acceptance of and Preference for COVID-19 Vaccination in India, the United Kingdom, Germany, Italy, and Spain: An International Cross-Sectional Study |
| Donin et al., 2022 | Outcome | Vaccine effectiveness | Efficacy^a^ | Factors Affecting Young Adults' Decision Making to Undergo COVID-19 Vaccination: A Patient Preference Study |
| Fu et al., 2020 | Outcome | Vaccine effectiveness | Vaccine efficacy^a^ | Acceptance of and preference for COVID-19 vaccination in healthcare workers: a comparative analysis and discrete choice experiment |
| Fung et al., 2022 | Outcome | Vaccine effectiveness | efficacy against COVID-19 infection^a^ | COVID-19 Vaccination Preferences of University Students and Staff in Hong Kong |
| Fung et al., 2022 | Outcome | Vaccine effectiveness | efficacy against severe manifestation of COVID-19 infection^a^ | COVID-19 Vaccination Preferences of University Students and Staff in Hong Kong |
| George et al., 2022 | Outcome | Vaccine effectiveness | Effectiveness^a^ | South African University Staff and Students' Perspectives, Preferences, and Drivers of Hesitancy Regarding COVID-19 Vaccines: A Multi-Methods Study |
| Hazlewood et al., 2023 | Outcome | Vaccine effectiveness | Vaccine Effectiveness^a^ | Preferences for COVID-19 Vaccination in People With Chronic Immune-Mediated Inflammatory Diseases |
| Huang et al., 2021 | Outcome | Vaccine effectiveness | vaccine effectiveness (95% vs 80%)^a^ | COVID-19 vaccine coverage, concerns, and preferences among Chinese ICU clinicians: a nationwide online survey |
| Krueger et al., 2022 | Outcome | Vaccine effectiveness | effectiveness^a^ | Stated choice analysis of preferences for COVID-19 vaccines using the Choquet integral |
| Leng et al., 2021 | Outcome | Vaccine effectiveness | vaccine effectiveness^a^ | Individual preferences for COVID-19 vaccination in China |
| Li et al., 2021 | Outcome | Vaccine effectiveness | effcacy^a^ | COVID-19 vaccine preferences among university students in Hong Kong: a discrete choice experiment |
| Li et al., 2023 | Outcome | Vaccine effectiveness | Efficacy^a^ | Understanding influencing attributes of COVID-19 vaccine preference and willingness-to-pay among Chinese and American middle-aged and elderly adults: A discrete choice experiment and propensity score matching study |
| Liu et al., 2021 | Outcome | Vaccine effectiveness | Efficacy^a^ | A Comparison of Vaccine Hesitancy of COVID-19 Vaccination in China and the United States |
| McPhedran et al., 2021 | Outcome | Vaccine effectiveness | Level of protection offered^a^ | Efficacy or delivery? An online Discrete Choice Experiment to explore preferences for COVID-19 vaccines in the UK |
| Morillon et al., 2022 | Outcome | Vaccine effectiveness | effectiveness^a^ | Public Preferences for a COVID-19 Vaccination Program in Quebec: A Discrete Choice Experiment |
| Mouter et al., 2022 | Outcome | Vaccine effectiveness | The percentage of vaccinated individuals protected against COVID-19^a^ | "Please, you go first!" preferences for a COVID-19 vaccine among adults in the Netherlands |
| Panchalingam et al., 2022 | Outcome | Vaccine effectiveness | Effectiveness^a^ | Parental refusal and hesitancy of vaccinating children against COVID-19: Findings from a nationally representative sample of parents in the U.S |
| Prosser et al., 2023 | Outcome | Vaccine effectiveness | Vaccine effectiveness^a^ | A Discrete Choice Analysis Comparing COVID-19 Vaccination Decisions for Children and Adults |
| Schwarzinger et al., 2021 | Outcome | Vaccine effectiveness | vaccine efficacy^a^ | COVID-19 vaccine hesitancy in a representative working-age population in France: a survey experiment based on vaccine characteristics |
| Teh et al., 2022 | Outcome | Vaccine effectiveness | Vaccine effectiveness^a^ | Malaysian public preferences and decision making for COVID-19 vaccination: A discrete choice experiment |
| Tran et al., 2023 | Outcome | Vaccine effectiveness | effectiveness^a^ | Preference and Willingness to Pay for the Regular COVID-19 Booster Shot in the Vietnamese Population: Theory-Driven Discrete Choice Experiment |
| Wang et al., 2021 | Outcome | Vaccine effectiveness | Probability of COVID-19 infection^a^ | Influence of Vaccination Characteristics on COVID-19 Vaccine Acceptance Among Working-Age People in Hong Kong, China: A Discrete Choice Experiment |
| Wang et al., 2022 | Outcome | Vaccine effectiveness | Efficacy ^a^ | Impact of information framing and vaccination characteristics on parental COVID-19 vaccine acceptance for children: a discrete choice experiment |
| Wang et al., 2022 | Outcome | Vaccine effectiveness | Vaccine effectiveness^a^ | Student COVID-19 vaccination preferences in China: A discrete choice experiment |
| Wang et al., 2022 | Outcome | Vaccine effectiveness | Vaccine effectiveness^a^ | Individual Preferences for COVID-19 Vaccination under the China's 2021 National Vaccination Policy: A Discrete Choice Experiment Study |
| Xiao et al., 2022 | Outcome | Vaccine effectiveness | Effectiveness^a^ | Attribute nonattendance in COVID-19 vaccine choice: A discrete choice experiment based on Chinese public preference |
| Zhang et al., 2022 | Outcome | Vaccine effectiveness | Efficacy^a^ | Personality Effects on Chinese Public Preference for the COVID-19 Vaccination: Discrete Choice Experiment and Latent Profile Analysis Study |
| Borriello et al., 2021 | Outcome | Vaccine effectiveness | Vaccination effectiveness^a^ | Location, location, location: a discrete choice experiment to inform COVID-19 vaccination programme delivery in the UK |
| Borriello et al., 2021 | Outcome | Vaccine effectiveness | Vaccine effectiveness^a^ | Vaccination or NPI? A conjoint analysis of German citizens' preferences in the context of the COVID-19 pandemic |
| Igarashi et al., 2022 | Outcome | Vaccine effectiveness | efficacy^a^ | Vaccination or NPI? A conjoint analysis of German citizens' preferences in the context of the COVID-19 pandemic |
| Igarashi et al., 2022 | Outcome | Vaccine effectiveness | Decrease deaths | Public Preferences for Policies to Promote COVID-19 Vaccination Uptake: A Discrete Choice Experiment in The Netherlands |
| Igarashi et al., 2022 | Outcome | Vaccine effectiveness | Decrease health damage | Public Preferences for Policies to Promote COVID-19 Vaccination Uptake: A Discrete Choice Experiment in The Netherlands |
| Igarashi et al., 2022 | Outcome | Vaccine effectiveness | Decrease households with income loss | Public Preferences for Policies to Promote COVID-19 Vaccination Uptake: A Discrete Choice Experiment in The Netherlands |
| Velardo et al., 2021 | Outcome | Vaccine effectiveness | Vaccine efficacy^a^ | How should COVID-19 vaccines be distributed between the Global North and South: a discrete choice experiment in six European countries |
| Wang et al., 2022 | Outcome | Vaccine effectiveness | Effectiveness (%)^a^ | How should COVID-19 vaccines be distributed between the Global North and South: a discrete choice experiment in six European countries |
| Bansal et al., 2022 | Outcome | Protection duration | Duration of protection offered by the vaccine^a^ | COVID-19 vaccine preferences in India |
| Blaga et al., 2023 | Outcome | Protection duration | duration of protection provided by the vaccine^a^ | Examination of Preferences for COVID-19 Vaccines in Hungary Based on Their Properties-Examining the Impact of Pandemic Awareness with a Hybrid Choice Approach |
| Chen et al., 2023 | Outcome | Protection duration | protective duration^a^ | The COVID-19 vaccination decision-making preferences of elderly people: a discrete choice experiment |
| Chen et al., 2021 | Outcome | protection duration | protection duration^a^ | Public preference and vaccination willingness for COVID-19 vaccine in China |
| Craig, 2021 | Outcome | protection duration | Duration of immunity^a^ | United States COVID-19 Vaccination Preferences (CVP): 2020 Hindsight |
| Darrudi et al., 2022 | Outcome | Protection duration | duration of protection^a^ | Public Preferences and Willingness to Pay for a COVID-19 Vaccine in Iran: A Discrete Choice Experiment |
| Daziano et al. 2022 | Outcome | Protection duration | Duration of protection^a^ | A choice experiment assessment of stated early response to COVID-19 vaccines in the USA |
| Díaz Luévano et al., 2021 | Outcome | Protection duration | indirect protection^a^ | Quantifying healthcare and welfare sector workers' preferences around COVID-19 vaccination: a cross-sectional, single-profile discrete-choice experiment in France |
| Díaz Luévano et al., 2021 | Outcome | Protection duration | protection duration^a^ | Quantifying healthcare and welfare sector workers' preferences around COVID-19 vaccination: a cross-sectional, single-profile discrete-choice experiment in France |
| Dong et al., 2020 | Outcome | Protection duration | Duration of protection (month)^a^ | Public preference for COVID-19 vaccines in China: A discrete choice experiment |
| Dong et al., 2022 | Outcome | Protection duration | the duration of the vaccine^a^ | Acceptance of and Preference for COVID-19 Vaccination in India, the United Kingdom, Germany, Italy, and Spain: An International Cross-Sectional Study |
| Donin et al., 2022 | Outcome | Protection duration | Protection duration^a^ | Factors Affecting Young Adults' Decision Making to Undergo COVID-19 Vaccination: A Patient Preference Study |
| Fung et al., 2022 | Outcome | Protection duration | duration of protection after vaccination^a^ | COVID-19 Vaccination Preferences of University Students and Staff in Hong Kong |
| Huang et al., 2021 | Outcome | Protection duration | duration of immunity (6 vs 12 months)^a^ | COVID-19 vaccine coverage, concerns, and preferences among Chinese ICU clinicians: a nationwide online survey |
| Krueger et al., 2022 | Outcome | Protection duration | protection period^a^ | Stated choice analysis of preferences for COVID-19 vaccines using the Choquet integral |
| Leng et al., 2021 | Outcome | Protection duration | duration of vaccine protection^a^ | Individual preferences for COVID-19 vaccination in China |
| Li et al., 2021 | Outcome | Protection duration | protect duration | COVID-19 vaccine preferences among university students in Hong Kong: a discrete choice experiment |
| Liu et al., 2021 | Outcome | Protection duration | The duration of vaccine effectiveness^a^ | A Comparison of Vaccine Hesitancy of COVID-19 Vaccination in China and the United States |
| Morillon et al., 2022 | Outcome | Protection duration | duration^a^ | Public Preferences for a COVID-19 Vaccination Program in Quebec: A Discrete Choice Experiment |
| Panchalingam et al., 2022 | Outcome | Protection duration | Duration of vaccine-induced protection^a^ | Parental refusal and hesitancy of vaccinating children against COVID-19: Findings from a nationally representative sample of parents in the U.S |
| Tran et al., 2023 | Outcome | Protection duration | immunity duration^a^ | Preference and Willingness to Pay for the Regular COVID-19 Booster Shot in the Vietnamese Population: Theory-Driven Discrete Choice Experiment |
| Wang et al., 2022 | Outcome | Protection duration | Duration of vaccine protection^a^ | Student COVID-19 vaccination preferences in China: A discrete choice experiment |
| Wang et al., 2022 | Outcome | Protection duration | duration of protection^a^ | Individual Preferences for COVID-19 Vaccination under the China's 2021 National Vaccination Policy: A Discrete Choice Experiment Study |
| Xiao et al., 2022 | Outcome | Protection duration | protection period^a^ | Attribute nonattendance in COVID-19 vaccine choice: A discrete choice experiment based on Chinese public preference |
| Zhang et al., 2022 | Outcome | Protection duration | Duration^a^ | Personality Effects on Chinese Public Preference for the COVID-19 Vaccination: Discrete Choice Experiment and Latent Profile Analysis Study |
| Hess et al., 2022 | Outcome | Protection duration | Estimated protection duration | Vaccination or NPI? A conjoint analysis of German citizens' preferences in the context of the COVID-19 pandemic |
| Igarashi et al., 2022 | Outcome | Protection duration | immunity duration^a^ | Vaccination or NPI? A conjoint analysis of German citizens' preferences in the context of the COVID-19 pandemic |
| Velardo et al., 2021 | Outcome | Protection duration | Duration of vaccine immunity^a^ | How should COVID-19 vaccines be distributed between the Global North and South: a discrete choice experiment in six European countries |
| Daziano, 2022 | Outcome | Time to vaccine efficacy | Days for antibodies to develop^a^ | A choice experiment assessment of stated early response to COVID-19 vaccines in the USA |
| Dong et al., 2022 | Outcome | Time to vaccine efficacy | time taken for the vaccine to work^a^ | Acceptance of and Preference for COVID-19 Vaccination in India, the United Kingdom, Germany, Italy, and Spain: An International Cross-Sectional Study |
| Krueger et al., 2022 | Outcome | Time to vaccine efficacy | incubation period^a^ | Stated choice analysis of preferences for COVID-19 vaccines using the Choquet integral |
| Li et al., 2023 | Outcome | Time to vaccine efficacy | The duration of vaccine works^a^ | Understanding influencing attributes of COVID-19 vaccine preference and willingness-to-pay among Chinese and American middle-aged and elderly adults: A discrete choice experiment and propensity score matching study |
| Liu et al., 2021 | Outcome | Time to vaccine efficacy | Time for the vaccine to start working | A Comparison of Vaccine Hesitancy of COVID-19 Vaccination in China and the United States |
| Zhang et al., 2022 | Outcome | Time to vaccine efficacy | Start working^a^ | Personality Effects on Chinese Public Preference for the COVID-19 Vaccination: Discrete Choice Experiment and Latent Profile Analysis Study |
| Asim et al., 2023 | Progress | Vaccine production | vaccine brand^a^ | COVID-19 Vaccination Preferences Among Non-Chinese Migrants in Hong Kong: Discrete Choice Experiment |
| Bansal et al., 2022 | Progress | Vaccine production | Developer ^a^ | COVID-19 vaccine preferences in India |
| Blaga et al., 2023 | Progress | Vaccine production | country of origin^a^ | Examination of Preferences for COVID-19 Vaccines in Hungary Based on Their Properties-Examining the Impact of Pandemic Awareness with a Hybrid Choice Approach |
| Darrudi et al., 2022 | Progress | Vaccine production | location of vaccine production^a^ | Public Preferences and Willingness to Pay for a COVID-19 Vaccine in Iran: A Discrete Choice Experiment |
| Daziano, 2022 | Progress | Vaccine production | Country where vaccine was developed^a^, | A choice experiment assessment of stated early response to COVID-19 vaccines in the USA |
| Daziano, 2022 | Progress | Vaccine production | Introduced [months]^a^ | A choice experiment assessment of stated early response to COVID-19 vaccines in the USA |
| Dong et al., 2020 | Progress | Vaccine production | Origin of product^a^ | Public preference for COVID-19 vaccines in China: A discrete choice experiment |
| Dong et al., 2022 | Progress | Vaccine production | vaccine types^a^ | Acceptance of and Preference for COVID-19 Vaccination in India, the United Kingdom, Germany, Italy, and Spain: An International Cross-Sectional Study |
| George et al., 2022 | Progress | Vaccine production | Vaccine origin^a^ | South African University Staff and Students' Perspectives, Preferences, and Drivers of Hesitancy Regarding COVID-19 Vaccines: A Multi-Methods Study |
| Krueger et al., 2022 | Progress | Vaccine production | origin of the vaccine^a^ | Stated choice analysis of preferences for COVID-19 vaccines using the Choquet integral |
| Li et al., 2021 | Progress | Vaccine production | origin of the vaccine^a^ | COVID-19 vaccine preferences among university students in Hong Kong: a discrete choice experiment |
| Li et al., 2023 | Progress | Vaccine production | Vaccine varieties^a^ | Understanding influencing attributes of COVID-19 vaccine preference and willingness-to-pay among Chinese and American middle-aged and elderly adults: A discrete choice experiment and propensity score matching study |
| Liu et al., 2021 | Progress | Vaccine production | Vaccine varieties^a^ | A Comparison of Vaccine Hesitancy of COVID-19 Vaccination in China and the United States |
| Morillon et al., 2022 | Progress | Vaccine production | origin^a^ | Public Preferences for a COVID-19 Vaccination Program in Quebec: A Discrete Choice Experiment |
| Schwarzinger et al., 2021 | Progress | Vaccine production | country of vaccine manufacturer^a^ | COVID-19 vaccine hesitancy in a representative working-age population in France: a survey experiment based on vaccine characteristics |
| Teh et al., 2022 | Progress | Vaccine production | Halal content^a^ | Malaysian public preferences and decision making for COVID-19 vaccination: A discrete choice experiment |
| Wang et al., 2021 | Progress | Vaccine production | Brand^a^ | Influence of Vaccination Characteristics on COVID-19 Vaccine Acceptance Among Working-Age People in Hong Kong, China: A Discrete Choice Experiment |
| Wang et al., 2022 | Progress | Vaccine production | Brand^a^ | Impact of information framing and vaccination characteristics on parental COVID-19 vaccine acceptance for children: a discrete choice experiment |
| Zhang et al., 2022 | Progress | Vaccine production | Varieties^a^ | Personality Effects on Chinese Public Preference for the COVID-19 Vaccination: Discrete Choice Experiment and Latent Profile Analysis Study |
| Velardo et al., 2021 | Progress | Vaccine production | Location of vaccine manufacturer^a^ | How should COVID-19 vaccines be distributed between the Global North and South: a discrete choice experiment in six European countries |
| Wang et al., 2022 | Progress | Vaccine production | Origin of Vaccine^a^ | How should COVID-19 vaccines be distributed between the Global North and South: a discrete choice experiment in six European countries |
| Asim et al., 2023 | Progress | Service delivery | venue for vaccination^a^ | COVID-19 Vaccination Preferences Among Non-Chinese Migrants in Hong Kong: Discrete Choice Experiment |
| Bansal et al., 2022 | Progress | Service delivery | Place where vaccination is administered^a^ | COVID-19 vaccine preferences in India |
| Craig et al. 2021 | Progress | Service delivery | Vaccination setting^a^ | United States COVID-19 Vaccination Preferences (CVP): 2020 Hindsight |
| Donin et al., 2022 | Progress | Service delivery | Vaccination location | Factors Affecting Young Adults' Decision Making to Undergo COVID-19 Vaccination: A Patient Preference Study |
| Donin et al., 2022 | Progress | Service delivery | Vaccination appointment scheduling | Factors Affecting Young Adults' Decision Making to Undergo COVID-19 Vaccination: A Patient Preference Study |
| George et al., 2022 | Progress | Service delivery | Vaccination location^a^ | South African University Staff and Students' Perspectives, Preferences, and Drivers of Hesitancy Regarding COVID-19 Vaccines: A Multi-Methods Study |
| Leng et al., 2021 | Progress | Service delivery | vaccination sites^a^ | Individual preferences for COVID-19 vaccination in China |
| Liu et al., 2021 | Progress | Service delivery | delivery mode^a^ | A Comparison of Vaccine Hesitancy of COVID-19 Vaccination in China and the United States |
| McPhedran et al., 2021 | Progress | Service delivery | Location in which the vaccine is administered^a^ | Efficacy or delivery? An online Discrete Choice Experiment to explore preferences for COVID-19 vaccines in the UK |
| Schwarzinger et al., 2021 | Progress | Service delivery | place to be vaccinated^a^ | COVID-19 vaccine hesitancy in a representative working-age population in France: a survey experiment based on vaccine characteristics |
| Teh et al., 2022 | Progress | Service delivery | vaccination schedule during office hours^a^ | Malaysian public preferences and decision making for COVID-19 vaccination: A discrete choice experiment |
| Wang et al., 2021 | Progress | Service delivery | Venue for vaccination^a^ | Influence of Vaccination Characteristics on COVID-19 Vaccine Acceptance Among Working-Age People in Hong Kong, China: A Discrete Choice Experiment |
| Wang et al., 2022 | Progress | Service delivery | Venue for vaccination | Impact of information framing and vaccination characteristics on parental COVID-19 vaccine acceptance for children: a discrete choice experiment |
| Wang et al., 2022 | Progress | Service delivery | Vaccination sites^a^ | Student COVID-19 vaccination preferences in China: A discrete choice experiment |
| Wang et al., 2022 | Progress | Service delivery | Vaccination sites^a^ | Individual Preferences for COVID-19 Vaccination under the China's 2021 National Vaccination Policy: A Discrete Choice Experiment Study |
| Borriello et al., 2021 | Progress | Service delivery | Location^a^ | Location, location, location: a discrete choice experiment to inform COVID-19 vaccination programme delivery in the UK |
| Igarashi et al., 2022 | progress | Service delivery | Vaccination at home | Public Preferences for Policies to Promote COVID-19 Vaccination Uptake: A Discrete Choice Experiment in The Netherlands |
| Igarashi et al., 2022 | progress | Service delivery | Vaccination when and where convenient | Public Preferences for Policies to Promote COVID-19 Vaccination Uptake: A Discrete Choice Experiment in The Netherlands |
| Velardo et al., 2021 | Progress | Service delivery | Place of vaccine administration | How should COVID-19 vaccines be distributed between the Global North and South: a discrete choice experiment in six European countries |
| Wang et al., 2022 | Progress | Service delivery | Location of vaccination^a^ | How should COVID-19 vaccines be distributed between the Global North and South: a discrete choice experiment in six European countries |
| Chen et al., 2023 | Progress | Dosing and visits | injection doses^a^ | The COVID-19 vaccination decision-making preferences of elderly people: a discrete choice experiment |
| Chen et al., 2023 | Progress | Dosing and visits | injection period^a^ | The COVID-19 vaccination decision-making preferences of elderly people: a discrete choice experiment |
| Dong et al., 2020 | Progress | Dosing and visits | The total number of injections^a^ | Public preference for COVID-19 vaccines in China: A discrete choice experiment |
| Donin et al., 2022 | Progress | Dosing and visits | Vaccine frequency | Factors Affecting Young Adults' Decision Making to Undergo COVID-19 Vaccination: A Patient Preference Study |
| Donin et al., 2022 | Progress | Dosing and visits | Number of doses required per vaccination episode | Factors Affecting Young Adults' Decision Making to Undergo COVID-19 Vaccination: A Patient Preference Study |
| George et al., 2022 | Progress | Dosing and visits | Boosters required^a^ | South African University Staff and Students' Perspectives, Preferences, and Drivers of Hesitancy Regarding COVID-19 Vaccines: A Multi-Methods Study |
| George et al., 2022 | Progress | Dosing and visits | Number of doses^a^ | South African University Staff and Students' Perspectives, Preferences, and Drivers of Hesitancy Regarding COVID-19 Vaccines: A Multi-Methods Study |
| Hazlewood et al., 2023 | Progress | Dosing and visits | Dosing^a^ | Preferences for COVID-19 Vaccination in People With Chronic Immune-Mediated Inflammatory Diseases |
| Krueger et al., 2022 | Progress | Dosing and visits | whether the vaccine has a booster against variants | Stated choice analysis of preferences for COVID-19 vaccines using the Choquet integral |
| Krueger et al., 2022 | Progress | Dosing and visits | number of required doses^a^ | Stated choice analysis of preferences for COVID-19 vaccines using the Choquet integral |
| Leng et al., 2021 | Progress | Dosing and visits | number of doses^a^ | Individual preferences for COVID-19 vaccination in China |
| Li et al., 2021 | Progress | Dosing and visits | required number of doses^a^ | COVID-19 vaccine preferences among university students in Hong Kong: a discrete choice experiment |
| McPhedran et al., 2021 | Progress | Dosing and visits | Number of doses needed for full protection^a^ | Efficacy or delivery? An online Discrete Choice Experiment to explore preferences for COVID-19 vaccines in the UK |
| Prosser et al., 2023 | Progress | Dosing and visits | number of doses^a^ | A Discrete Choice Analysis Comparing COVID-19 Vaccination Decisions for Children and Adults |
| Wang et al., 2022 | Progress | Dosing and visits | Number of doses^a^ | How should COVID-19 vaccines be distributed between the Global North and South: a discrete choice experiment in six European countries |
| Donin et al., 2022 | Progress | Time | Waiting time at vaccination site | Factors Affecting Young Adults' Decision Making to Undergo COVID-19 Vaccination: A Patient Preference Study |
| George et al., 2022 | Progress | Time | Waiting time at vaccination site^a^ | South African University Staff and Students' Perspectives, Preferences, and Drivers of Hesitancy Regarding COVID-19 Vaccines: A Multi-Methods Study |
| Liu et al., 2021 | Progress | Time | appointment timing^a^ | A Comparison of Vaccine Hesitancy of COVID-19 Vaccination in China and the United States |
| Morillon et al., 2022 | Progress | Time | waiting time^a^ | Public Preferences for a COVID-19 Vaccination Program in Quebec: A Discrete Choice Experiment |
| Mouter et al., 2022 | Progress | Time | The month in which the vaccine would become available to the respondent^a^ | "Please, you go first!" preferences for a COVID-19 vaccine among adults in the Netherlands |
| Prosser et al., 2023 | Progress | Time | total time required to get vaccinated^a^ | A Discrete Choice Analysis Comparing COVID-19 Vaccination Decisions for Children and Adults |
| Borriello et al., 2021 | Progress | Time | When available^a^ | Location, location, location: a discrete choice experiment to inform COVID-19 vaccination programme delivery in the UK |
| Borriello et al., 2021 | Progress | Time | Time of Covid vaccination^a^ | Vaccination or NPI? A conjoint analysis of German citizens' preferences in the context of the COVID-19 pandemic |
| Chen et al., 2021 | Progress | Vaccine accessibility | convenience of vaccination^a^ | Public preference and vaccination willingness for COVID-19 vaccine in China |
| Donin et al., 2022 | Progress | Vaccine accessibility | Travel time to vaccination site^a^ | Factors Affecting Young Adults' Decision Making to Undergo COVID-19 Vaccination: A Patient Preference Study |
| Leng et al., 2021 | Progress | Vaccine accessibility | accessibility^a^ | Individual preferences for COVID-19 vaccination in China |
| Liu et al., 2021 | Progress | Vaccine accessibility | proximity（travel time）^a^ | A Comparison of Vaccine Hesitancy of COVID-19 Vaccination in China and the United States |
| Teh et al., 2022 | Progress | Vaccine accessibility | distance from home to vaccination centre^a^ | Malaysian public preferences and decision making for COVID-19 vaccination: A discrete choice experiment |
| Darrudi et al., 2022 | Progress | priority population | Potential capacity to spread the virus (virus spread)^a^ | Public Preferences and Willingness to Pay for a COVID-19 Vaccine in Iran: A Discrete Choice Experiment |
| Darrudi et al., 2022 | Progress | priority population | Underlying disease^a^ | Public Preferences and Willingness to Pay for a COVID-19 Vaccine in Iran: A Discrete Choice Experiment |
| Morillon et al., 2022 | Progress | priority population | priority population^a^ | Public Preferences for a COVID-19 Vaccination Program in Quebec: A Discrete Choice Experiment |
| Igarashi et al., 2022 | Progress | priority population | Virus spreader^a^ | Rationing of a scarce life-saving resource: Public preferences for prioritizing COVID-19 vaccination |
| Igarashi et al., 2022 | Progress | priority population | mortality risk^a^ | How should COVID-19 vaccines be distributed between the Global North and South: a discrete choice experiment in six European countries |
| Igarashi et al., 2022 | Progress | priority population | Medical risk group^a^ | Rationing of a scarce life-saving resource: Public preferences for prioritizing COVID-19 vaccination |
| Igarashi et al., 2022 | Progress | priority population | Essential profession^a^ | Rationing of a scarce life-saving resource: Public preferences for prioritizing COVID-19 vaccination |
| Darrudi et al., 2022 | Progress | Vaccination age | Age | Public Preferences and Willingness to Pay for a COVID-19 Vaccine in Iran: A Discrete Choice Experiment |
| Igarashi et al., 2022 | Progress | Vaccination age | Age^a^ | Rationing of a scarce life-saving resource: Public preferences for prioritizing COVID-19 vaccination |
| Igarashi et al., 2022 | Progress | Vaccination age | age^a^ | How should COVID-19 vaccines be distributed between the Global North and South: a discrete choice experiment in six European countries |
| Donin et al., 2022 | Progress | Vaccine administration | Route of vaccination^a^ | Factors Affecting Young Adults' Decision Making to Undergo COVID-19 Vaccination: A Patient Preference Study |
| Borriello et al., 2021 | Progress | Vaccine administration | Mode of administration^a^ | Location, location, location: a discrete choice experiment to inform COVID-19 vaccination programme delivery in the UK |
| Prosser et al., 2023 | progress | vaccination approval | regulatory approval^a^ | A Discrete Choice Analysis Comparing COVID-19 Vaccination Decisions for Children and Adults |
| Bansal et al., 2022 | Cost | Out-of-pocket cost | Out-of-pocket cost^a^ | 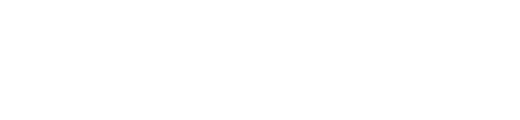COVID-19 vaccine preferences in India |
| Chen et al., 2021 | Cost | Out-of-pocket cost | out-of-pocket cost^a^ | Public preference and vaccination willingness for COVID-19 vaccine in China |
| Darrudi et al., 2022 | Cost | Out-of-pocket cost | price^a^ | 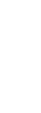Public Preferences and Willingness to Pay for a COVID-19 Vaccine in Iran: A Discrete Choice Experiment |
| Daziano, 2022 | Cost | Out-of-pocket cost | Out-of-pocket cost^a^ | A choice experiment assessment of stated early response to COVID-19 vaccines in the USA |
| Dong et al., 2020 | Cost | Out-of-pocket cost | Price(CNY)^a^ | Public preference for COVID-19 vaccines in China: A discrete choice experiment |
| Dong et al., 2022 | Cost | Out-of-pocket cost | the cost of vaccination^a^ | Acceptance of and Preference for COVID-19 Vaccination in India, the United Kingdom, Germany, Italy, and Spain: An International Cross-Sectional Study |
| Fu et al., 2020 | Cost | Out-of-pocket cost | Out of pocket of the vaccination^a^ | Acceptance of and preference for COVID-19 vaccination in healthcare workers: a comparative analysis and discrete choice experiment |
| Fung et al., 2022 | Cost | Out-of-pocket cost | out-of-pocket costs^a^ | COVID-19 Vaccination Preferences of University Students and Staff in Hong Kong |
| Krueger et al., 2022 | Cost | Out-of-pocket cost | out-of-pocket cost^a^ | Stated choice analysis of preferences for COVID-19 vaccines using the Choquet integral |
| Li et al., 2021 | Cost | Out-of-pocket cost | out-of-pocket price ^a^ | COVID-19 vaccine preferences among university students in Hong Kong: a discrete choice experiment |
| Li et al., 2023 | Cost | Out-of-pocket cost | The cost of vaccination^a^ | Understanding influencing attributes of COVID-19 vaccine preference and willingness-to-pay among Chinese and American middle-aged and elderly adults: A discrete choice experiment and propensity score matching study |
| Liu et al., 2021 | Cost | Out-of-pocket cost | The cost of vaccination^a^ | A Comparison of Vaccine Hesitancy of COVID-19 Vaccination in China and the United States |
| Tran et al., 2023 | Cost | Out-of-pocket cost | cost of the vaccine | Preference and Willingness to Pay for the Regular COVID-19 Booster Shot in the Vietnamese Population: Theory-Driven Discrete Choice Experiment |
| Xiao et al., 2022 | Cost | Out-of-pocket cost | price^a^ | Attribute nonattendance in COVID-19 vaccine choice: A discrete choice experiment based on Chinese public preference |
| Zhang et al., 2022 | Cost | Out-of-pocket cost | The cost^a^ | Personality Effects on Chinese Public Preference for the COVID-19 Vaccination: Discrete Choice Experiment and Latent Profile Analysis Study |
| Borriello et al., 2021 | Cost | Out-of-pocket cost | Cost^a^ | Location, location, location: a discrete choice experiment to inform COVID-19 vaccination programme delivery in the UK |
| Hess et al., 2022 | Cost | Out-of-pocket cost | Fee | Vaccination or NPI? A conjoint analysis of German citizens' preferences in the context of the COVID-19 pandemic |
| Igarashi et al., 2022 | Cost | Out-of-pocket cost | price^a^ | Vaccination or NPI? A conjoint analysis of German citizens' preferences in the context of the COVID-19 pandemic |
| Wang et al., 2022 | Cost | Out-of-pocket cost | Price(CNY)^a^ | How should COVID-19 vaccines be distributed between the Global North and South: a discrete choice experiment in six European countries |
| Darrudi et al., 2022 | Cost | Cost to society | Cost to the community^a^ | Public Preferences and Willingness to Pay for a COVID-19 Vaccine in Iran: A Discrete Choice Experiment |
| Igarashi et al., 2022 | Cost | Cost to society | Cost to society^a^ | Rationing of a scarce life-saving resource: Public preferences for prioritizing COVID-19 vaccination |
| Igarashi et al., 2022 | Cost | One-time tax increase | One-time tax increase^a^ | Public Preferences for Policies to Promote COVID-19 Vaccination Uptake: A Discrete Choice Experiment in The Netherlands |
| Asim et al., 2023 | Other | Incentives/penalties for vaccination | exemption of quarantine for vaccinated travelers^a^ | COVID-19 Vaccination Preferences Among Non-Chinese Migrants in Hong Kong: Discrete Choice Experiment |
| Craig, 2021 | Other | Incentives/penalties for vaccination | Proof of vaccination^a^ | United States COVID-19 Vaccination Preferences (CVP): 2020 Hindsight |
| Fung et al., 2022 | Other | Incentives/penalties for vaccination | incentive for completing vaccination^a^ | COVID-19 Vaccination Preferences of University Students and Staff in Hong Kong |
| George et al., 2022 | Other | Incentives/penalties for vaccination | Incentive for vaccination^a^ | South African University Staff and Students' Perspectives, Preferences, and Drivers of Hesitancy Regarding COVID-19 Vaccines: A Multi-Methods Study |
| Tran et al., 2023 | Other | Incentives/penalties for vaccination | limitations if not vaccinated^a^ | Preference and Willingness to Pay for the Regular COVID-19 Booster Shot in the Vietnamese Population: Theory-Driven Discrete Choice Experiment |
| Wang et al., 2021 | Other | Incentives/penalties for vaccination | Quarantine for vaccinated travelers^a^ | Influence of Vaccination Characteristics on COVID-19 Vaccine Acceptance Among Working-Age People in Hong Kong, China: A Discrete Choice Experiment |
| Borriello et al., 2021 | Other | Incentives/penalties for vaccination | Vaccination advantages/penalties^a^ | Vaccination or NPI? A conjoint analysis of German citizens' preferences in the context of the COVID-19 pandemic |
| Hess et al., 2022 | Other | Incentives/penalties for vaccination | Exemption from international travel restrictions | Vaccination or NPI? A conjoint analysis of German citizens' preferences in the context of the COVID-19 pandemic |
| Igarashi et al., 2022 | Other | Incentives/penalties for vaccination | Pay €250 if does not get vaccinated^a^ | Public Preferences for Policies to Promote COVID-19 Vaccination Uptake: A Discrete Choice Experiment in The Netherlands |
| Igarashi et al., 2022 | Other | Incentives/penalties for vaccination | Receive €100 if gets vaccinated^a^ | Public Preferences for Policies to Promote COVID-19 Vaccination Uptake: A Discrete Choice Experiment in The Netherlands |
| Igarashi et al., 2022 | Other | Incentives/penalties for vaccination | Vaccination passport daily activities during outbreak^a^ | Public Preferences for Policies to Promote COVID-19 Vaccination Uptake: A Discrete Choice Experiment in The Netherlands |
| Igarashi et al., 2022 | Other | Incentives/penalties for vaccination | Vaccination passport large events^a^ | Public Preferences for Policies to Promote COVID-19 Vaccination Uptake: A Discrete Choice Experiment in The Netherlands |
| Igarashi et al., 2022 | Other | Incentives/penalties for vaccination | Mandatory testing at own cost if does not get vaccinated^a^ | Public Preferences for Policies to Promote COVID-19 Vaccination Uptake: A Discrete Choice Experiment in The Netherlands |
| Asim et al., 2023 | Other | vaccination status of the public | vaccine by people around | COVID-19 Vaccination Preferences Among Non-Chinese Migrants in Hong Kong: Discrete Choice Experiment |
| Bansal et al., 2022 | Other | vaccination status of the public | The proportion of friends and family members who have taken the vaccine^a^ | COVID-19 vaccine preferences in India |
| Donin et al., 2022 | Other | vaccination status of the public | Who has already received the vaccine in your community? | Factors Affecting Young Adults' Decision Making to Undergo COVID-19 Vaccination: A Patient Preference Study |
| Fu et al., 2020 | Other | vaccination status of the public | Acceptance of social contacts^a^ | Acceptance of and preference for COVID-19 vaccination in healthcare workers: a comparative analysis and discrete choice experiment |
| Huang et al., 2021 | Other | vaccination status of the public | whether coworkers have been vaccinated^a^ | COVID-19 vaccine coverage, concerns, and preferences among Chinese ICU clinicians: a nationwide online survey |
| Leng et al., 2021 | Other | vaccination status of the public | proportion of acquaintances vaccinated^a^ | Individual preferences for COVID-19 vaccination in China |
| Panchalingam et al., 2022 | Other | vaccination status of the public | Local coverage^a^ | Parental refusal and hesitancy of vaccinating children against COVID-19: Findings from a nationally representative sample of parents in the U.S |
| Wang et al., 2021 | Other | vaccination status of the public | Vaccine uptake of people around^a^ | Influence of Vaccination Characteristics on COVID-19 Vaccine Acceptance Among Working-Age People in Hong Kong, China: A Discrete Choice Experiment |
| Wang et al., 2022 | Other | vaccination status of the public | Vaccination coverage among all children under 18 years^a^ | Impact of information framing and vaccination characteristics on parental COVID-19 vaccine acceptance for children: a discrete choice experiment |
| Wang et al., 2022 | Other | vaccination status of the public | Vaccine uptake among acquaintances’ minor children | Impact of information framing and vaccination characteristics on parental COVID-19 vaccine acceptance for children: a discrete choice experiment |
| Wang et al., 2022 | Other | vaccination status of the public | Acquaintances vaccinated^a^ | Student COVID-19 vaccination preferences in China: A discrete choice experiment |
| Wang et al., 2022 | Other | vaccination status of the public | percentage of acquaintances vaccinated^a^ | Individual Preferences for COVID-19 Vaccination under the China's 2021 National Vaccination Policy: A Discrete Choice Experiment Study |
| Hess et al., 2022 | Other | vaccination status of the public | Population coverage | Vaccination or NPI? A conjoint analysis of German citizens' preferences in the context of the COVID-19 pandemic |
| Fu et al., 2020 | Other | Disease risk | Infection probability^a^ | Acceptance of and preference for COVID-19 vaccination in healthcare workers: a comparative analysis and discrete choice experiment |
| Fu et al., 2020 | Other | Disease risk | Case-fatality ratio^a^ | Acceptance of and preference for COVID-19 vaccination in healthcare workers: a comparative analysis and discrete choice experiment |
| Fu et al., 2020 | Other | Disease risk | Possible trends of the epidemic^a^ | Acceptance of and preference for COVID-19 vaccination in healthcare workers: a comparative analysis and discrete choice experiment |
| Panchalingam et al., 2022 | Other | Disease risk | Risk of unvaccinated children requiring hospitalization for COVID-19^a^ | Parental refusal and hesitancy of vaccinating children against COVID-19: Findings from a nationally representative sample of parents in the U.S |
| Tran et al., 2023 | Other | Disease risk | COVID-19 mortality rate | Preference and Willingness to Pay for the Regular COVID-19 Booster Shot in the Vietnamese Population: Theory-Driven Discrete Choice Experiment |
| Wang et al., 2022 | Other | Disease risk | Risk perception^a^ | Student COVID-19 vaccination preferences in China: A discrete choice experiment |
| Wang et al., 2022 | Other | Disease risk | perceived probability of infection of individuals/acquaintances^a^ | Individual Preferences for COVID-19 Vaccination under the China's 2021 National Vaccination Policy: A Discrete Choice Experiment Study |
| Hess et al., 2022 | Other | Disease risk | Risk of infection | Vaccination or NPI? A conjoint analysis of German citizens' preferences in the context of the COVID-19 pandemic |
| Hess et al., 2022 | Other | Disease risk | Risk of serious illness | Vaccination or NPI? A conjoint analysis of German citizens' preferences in the context of the COVID-19 pandemic |
| Asim et al., 2023 | Other | Vaccine advice/support | uptake of recommendations from professionals | COVID-19 Vaccination Preferences Among Non-Chinese Migrants in Hong Kong: Discrete Choice Experiment |
| Daziano, 2022 | Other | Vaccine advice/support | Who recommends this specific vaccine^a^ | A choice experiment assessment of stated early response to COVID-19 vaccines in the USA |
| Díaz Luévano et al., 2021 | Other | Vaccine advice/support | recommendation/incentive source^a^ | Quantifying healthcare and welfare sector workers' preferences around COVID-19 vaccination: a cross-sectional, single-profile discrete-choice experiment in France |
| Donin et al., 2022 | Other | Vaccine advice/support | Recommender of the vaccine^a^ | Factors Affecting Young Adults' Decision Making to Undergo COVID-19 Vaccination: A Patient Preference Study |
| Liu et al., 2021 | Other | Vaccine advice/support | sender^a^ | A Comparison of Vaccine Hesitancy of COVID-19 Vaccination in China and the United States |
| McPhedran et al., 2021 | Other | Vaccine advice/support | Recommender of the vaccine^a^ | Efficacy or delivery? An online Discrete Choice Experiment to explore preferences for COVID-19 vaccines in the UK |
| Morillon et al., 2022 | Other | Vaccine advice/support | recommendation^a^ | Public Preferences for a COVID-19 Vaccination Program in Quebec: A Discrete Choice Experiment |
| Wang et al., 2021 | Other | Vaccine advice/support | Recommendations from professionals | Influence of Vaccination Characteristics on COVID-19 Vaccine Acceptance Among Working-Age People in Hong Kong, China: A Discrete Choice Experiment |
| Wang et al., 2022 | Other | Vaccine advice/support | Recommendations from professionals | Impact of information framing and vaccination characteristics on parental COVID-19 vaccine acceptance for children: a discrete choice experiment |
| Igarashi et al., 2022 | Other | Vaccine advice/support | Vaccination ambassadors | Public Preferences for Policies to Promote COVID-19 Vaccination Uptake: A Discrete Choice Experiment in The Netherlands |
| Igarashi et al., 2022 | Other | Vaccine advice/support | Counselling if does not get vaccinated^a^ | Public Preferences for Policies to Promote COVID-19 Vaccination Uptake: A Discrete Choice Experiment in The Netherlands |
| Darrudi et al., 2022 | Other | other | Employment in the health sector^a^, | Public Preferences and Willingness to Pay for a COVID-19 Vaccine in Iran: A Discrete Choice Experiment |
| Darrudi et al., 2022 | Other | other | The necessary job for society^a^ | Public Preferences and Willingness to Pay for a COVID-19 Vaccine in Iran: A Discrete Choice Experiment |
| McPhedran et al., 2021 | Other | other | Coverage in the media^a^ | Efficacy or delivery? An online Discrete Choice Experiment to explore preferences for COVID-19 vaccines in the UK |
| Igarashi et al., 2022 | Other | other | Employment status^a^ | How should COVID-19 vaccines be distributed between the Global North and South: a discrete choice experiment in six European countries |
| Igarashi et al., 2022 | Other | other | Country of residence and healthcare system capacity^a^ | How should COVID-19 vaccines be distributed between the Global North and South: a discrete choice experiment in six European countries |
| Borriello et al., 2021 | Other | Non-pharmaceutical interventions | Non-pharmaceutical interventions restriction level^a^ | Vaccination or NPI? A conjoint analysis of German citizens' preferences in the context of the COVID-19 pandemic |
| Borriello et al., 2021 | Other | Non-pharmaceutical interventions | Work site^a^ | Vaccination or NPI? A conjoint analysis of German citizens' preferences in the context of the COVID-19 pandemic |
| Donin et al., 2022 | Other | Vaccination enforcement | Vaccination enforcement | Factors Affecting Young Adults' Decision Making to Undergo COVID-19 Vaccination: A Patient Preference Study |
| Borriello et al., 2021 | Other | Vaccination enforcement | Choices to get vaccinated^a^ | Vaccination or NPI? A conjoint analysis of German citizens' preferences in the context of the COVID-19 pandemic |

**DCE, Discrete Choice Experiments**

^a^ Attribute is significant *p* < 0.05
