# Supplementary material for: Preferences for COVID-19 Vaccines: Systematic Literature Review of Discrete Choice Experiments
Source: JMIR Public Health Surveill. 2024 Jul 29;10:e56546. doi: 10.2196/56546 (PMC11319885; doi:10.2196/56546)
Supplement: Multimedia Appendix 4 [file publichealth_v10i1e56546_app4.docx]

**Multimedia Appendix 4.** The detailed distribution of the study period across countries.


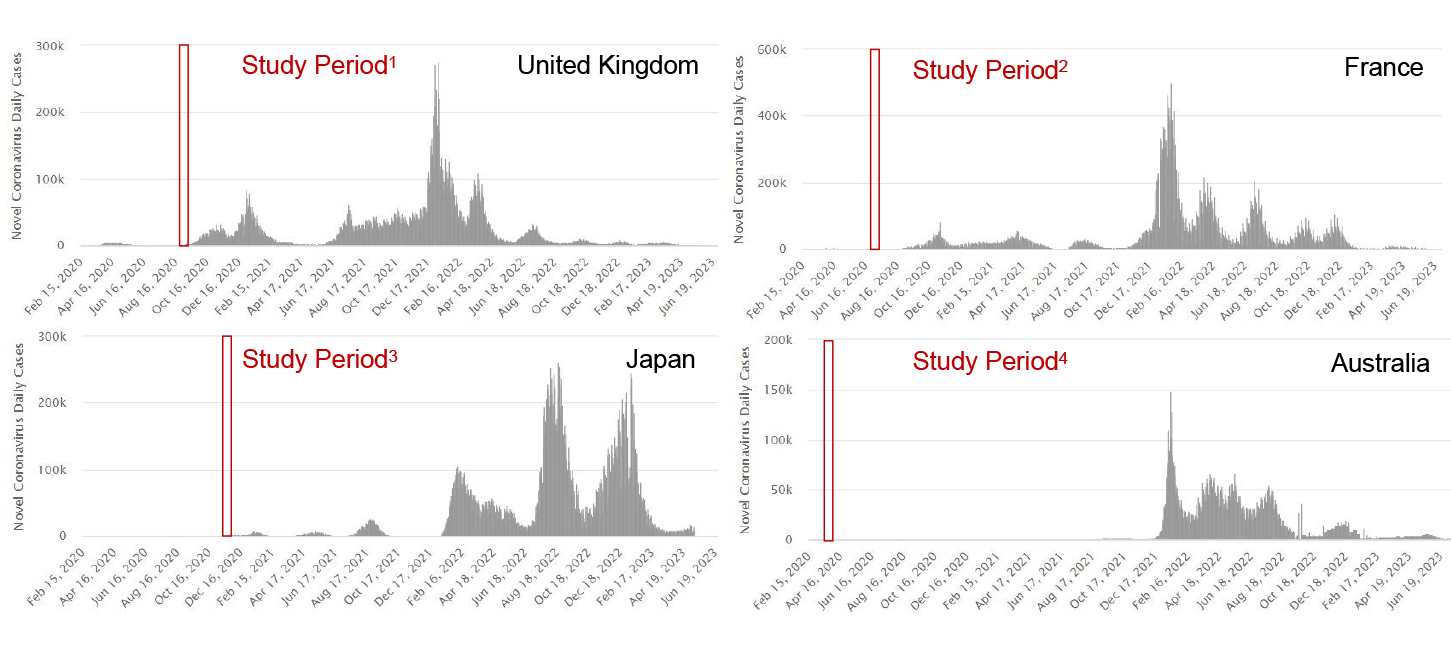


**4.1.** Before the pandemic.

**
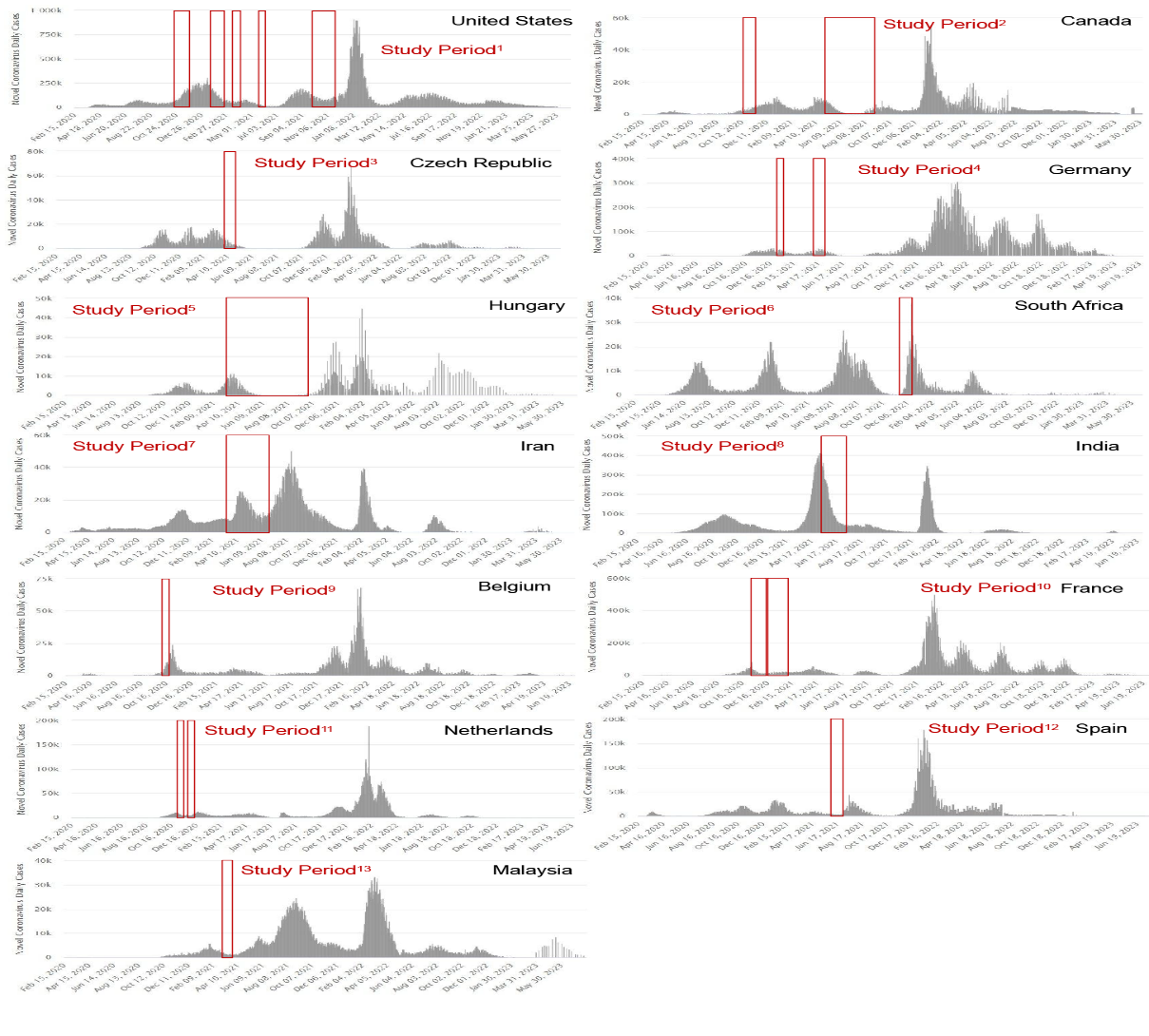
**

**4.2.** During the pandemic.

**
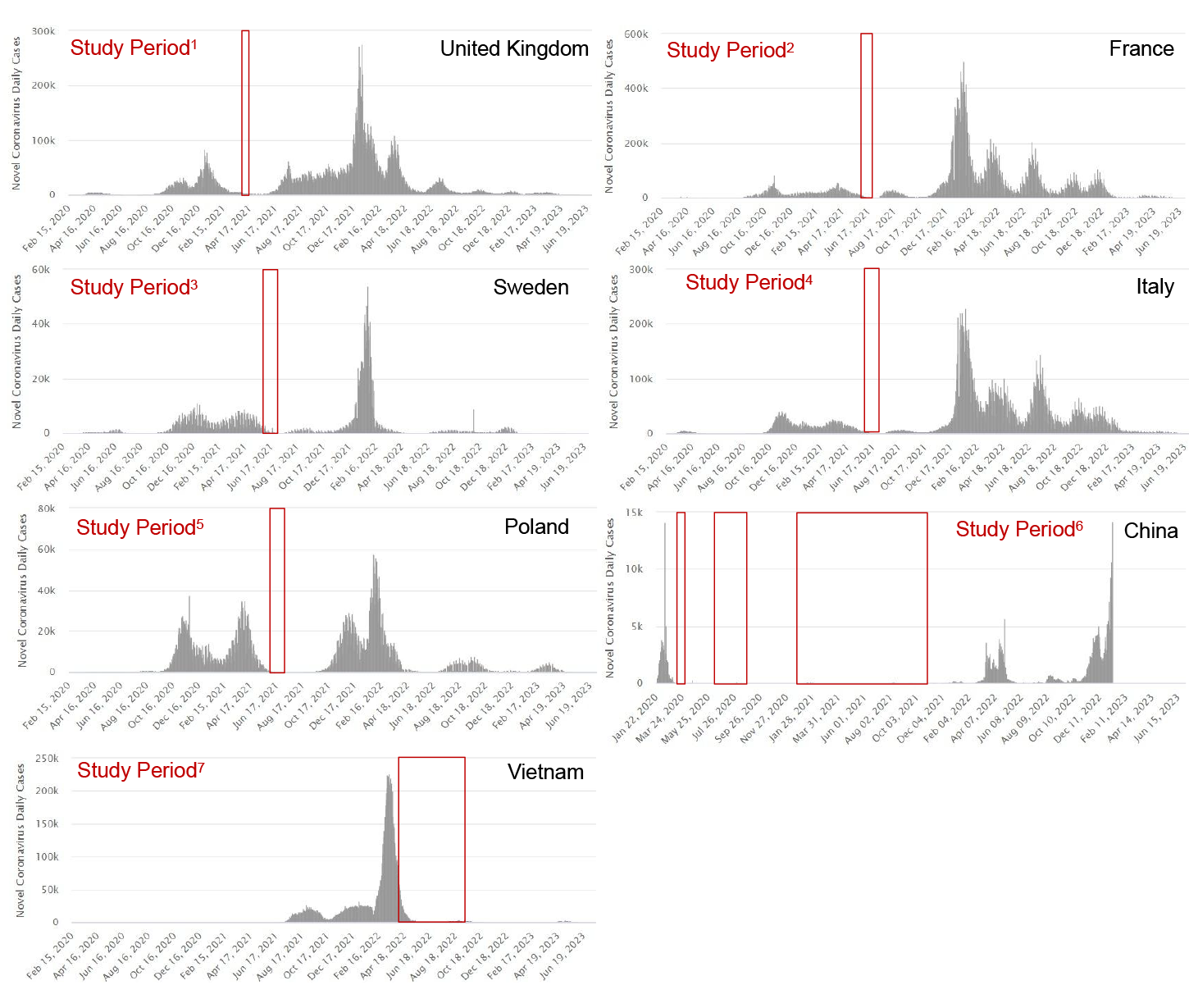
**

**4.3.** After the pandemic.

**4.4 Detailed information of the studies in different periods**

| Periods | | Author, et.al., Year | Title |
| --- | --- | --- | --- |
| Before the pandemic | | | |
|  | Study period 1 | McPhedran et al,. 2021 | Efficacy or delivery? An online Discrete Choice Experiment to explore preferences for COVID-19 vaccines in the UK |
|  | Study period 2 | Schwarzinger et al,. 2021 | COVID-19 vaccine hesitancy in a representative working-age population in France: a survey experiment based on vaccine characteristics |
|  | Study period 3 | Igarashi et al,. 2022 | Public preferences and willingness to accept a hypothetical vaccine to prevent a pandemic in Japan: a conjoint analysis |
|  | Study period 4 | Borriello et al,. 2021 | Preferences for a COVID-19 vaccine in Australia |
| During the pandemic | | | |
|  | Study period 1 | Craig, 2021 | United States COVID-19 Vaccination Preferences (CVP): 2020 Hindsight |
|  |  | Daziano, 2022 | A choice experiment assessment of stated early response to COVID-19 vaccines in the USA |
|  |  | Krueger et al,. 2022 | Stated choice analysis of preferences for COVID-19 vaccines using the Choquet integral |
|  |  | Panchalingam et al,. 2022 | Parental refusal and hesitancy of vaccinating children against COVID-19: Findings from a nationally representative sample of parents in the U.S |
|  |  | Li et al,. 2023 | Understanding influencing attributes of COVID-19 vaccine preference and willingness-to-pay among Chinese and American middle-aged and elderly adults: A discrete choice experiment and propensity score matching study |
|  |  | Liu et al,. 2021 | A Comparison of Vaccine Hesitancy of COVID-19 Vaccination in China and the United States |
|  |  | Prosser et al,. 2023 | A Discrete Choice Analysis Comparing COVID-19 Vaccination Decisions for Children and Adults |
|  |  | Eshun-Wilson et al,. 2021 | Preferences for COVID-19 vaccine distribution strategies in the US: A discrete choice survey |
|  | Study period 2 | Hazlewood et al,. 2023 | Preferences for COVID-19 Vaccination in People With Chronic Immune-Mediated Inflammatory Diseases |
|  | Study period 3 | Donin et al,. 2022 | Factors Affecting Young Adults' Decision Making to Undergo COVID-19 Vaccination: A Patient Preference Study |
|  |  | Morillon et al,. 2022 | Public Preferences for a COVID-19 Vaccination Program in Quebec: A Discrete Choice Experiment |
|  | Study period 4 | Bughin et al,. 2023 | Vaccination or NPI? A conjoint analysis of German citizens' preferences in the context of the COVID-19 pandemic |
|  |  | Steinert et al,. 2022 | How should COVID-19 vaccines be distributed between the Global North and South: a discrete choice experiment in six European countries |
|  | Study period 5 | Blaga et al,. 2023 | Examination of Preferences for COVID-19 Vaccines in Hungary Based on Their Properties-Examining the Impact of Pandemic Awareness with a Hybrid Choice Approach |
|  | Study period 6 | George et al,. 2022 | South African University Staff and Students' Perspectives, Preferences, and Drivers of Hesitancy Regarding COVID-19 Vaccines: A Multi-Methods Study |
|  | Study period 7 | Darrudi et al,. 2022 | Public Preferences and Willingness to Pay for a COVID-19 Vaccine in Iran: A Discrete Choice Experiment |
|  | Study period 8 | Bansal et al,. 2022 | COVID-19 vaccine preferences in India |
|  | Study period 9 | Luyten et al,. 2022 | Rationing of a scarce life-saving resource: Public preferences for prioritizing COVID-19 vaccination |
|  | Study period 10 | Díaz Luévano et al,. 2021 | Quantifying healthcare and welfare sector workers' preferences around COVID-19 vaccination: a cross-sectional, single-profile discrete-choice experiment in France |
|  |  | Velardo et al,. 2021 | Regional differences in COVID-19 vaccine hesitancy in december 2020: A natural experiment in the French working-age population |
|  | Study period 11 | Mouter et al,. 2022 | "Please, you go first!" preferences for a COVID-19 vaccine among adults in the Netherlands |
|  |  | Mouter et al,. 2022 | Public Preferences for Policies to Promote COVID-19 Vaccination Uptake: A Discrete Choice Experiment in The Netherlands |
|  | Study period 12 | Steinert et al,. 2022 | How should COVID-19 vaccines be distributed between the Global North and South: a discrete choice experiment in six European countries |
|  | Study period 13 | Teh et al,. 2022 | Malaysian public preferences and decision making for COVID-19 vaccination: A discrete choice experiment |
| After the pandemic | | | |
|  | Study period 1 | McPhedran et al,. 2022 | Location, location, location: a discrete choice experiment to inform COVID-19 vaccination programme delivery in the UK |
|  | Study period 2-5 | Steinert et al,. 2022 | How should COVID-19 vaccines be distributed between the Global North and South: a discrete choice experiment in six European countries |
|  | Study period 6 | Asim et al,. 2023 | COVID-19 Vaccination Preferences Among Non-Chinese Migrants in Hong Kong: Discrete Choice Experiment |
|  |  | Chen et al,. 2023 | The COVID-19 vaccination decision-making preferences of elderly people: a discrete choice experiment |
|  |  | Chen et al,. 2021 | Public preference and vaccination willingness for COVID-19 vaccine in China |
|  |  | Dong et al,. 2020 | Public preference for COVID-19 vaccines in China: A discrete choice experiment |
|  |  | Fu et al,. 2020 | Acceptance of and preference for COVID-19 vaccination in healthcare workers: a comparative analysis and discrete choice experiment |
|  |  | Fung et al,. 2022 | COVID-19 Vaccination Preferences of University Students and Staff in Hong Kong |
|  |  | Huang et al,. 2021 | COVID-19 vaccine coverage, concerns, and preferences among Chinese ICU clinicians: a nationwide online survey |
|  |  | Li et al,. 2021 | COVID-19 vaccine preferences among university students in Hong Kong: a discrete choice experiment |
|  |  | Li et al,. 2023 | Understanding influencing attributes of COVID-19 vaccine preference and willingness-to-pay among Chinese and American middle-aged and elderly adults: A discrete choice experiment and propensity score matching study |
|  |  | Liu et al,. 2021 | A Comparison of Vaccine Hesitancy of COVID-19 Vaccination in China and the United States |
|  |  | Wang et al,. 2021 | Influence of Vaccination Characteristics on COVID-19 Vaccine Acceptance Among Working-Age People in Hong Kong, China: A Discrete Choice Experiment |
|  |  | Wang et al,. 2022 | Impact of information framing and vaccination characteristics on parental COVID-19 vaccine acceptance for children: a discrete choice experiment |
|  |  | Wang et al,. 2022 | Student COVID-19 vaccination preferences in China: A discrete choice experiment |
|  |  | Wang et al,. 2022 | Individual Preferences for COVID-19 Vaccination under the China's 2021 National Vaccination Policy: A Discrete Choice Experiment Study |
|  |  | Xiao et al,. 2022 | Attribute nonattendance in COVID-19 vaccine choice: A discrete choice experiment based on Chinese public preference |
|  |  | Zhang et al,. 2022 | Personality Effects on Chinese Public Preference for the COVID-19 Vaccination: Discrete Choice Experiment and Latent Profile Analysis Study |
|  |  | Wang et al,. 2022 | Would COVID-19 vaccination willingness increase if mobile technologies prohibit unvaccinated individuals from public spaces? A nationwide discrete choice experiment from China |
|  | Study period 7 | Tran et al,. 2023 | Preference and Willingness to Pay for the Regular COVID-19 Booster Shot in the Vietnamese Population: Theory-Driven Discrete Choice Experiment |
