# Supplementary material for: Preferences for COVID-19 Vaccines: Systematic Literature Review of Discrete Choice Experiments
Source: JMIR Public Health Surveill. 2024 Jul 29;10:e56546. doi: 10.2196/56546 (PMC11319885; doi:10.2196/56546)
Supplement: Multimedia Appendix 5 [file publichealth_v10i1e56546_app5.docx]

**Multimedia Appendix 5. Preference for COVID-19 vaccines among high-income countries and low- and middle-income countries(N=53)**

| **Most important attributes** | **Number** | **%** |
| --- | --- | --- |
| **High-income countries (N=29)** |  |  |
| Effectiveness | 11 | 38 |
| Mortality risk | 6 | 21 |
| Safety | 3 | 10 |
| Protection duration | 2 | 7 |
| SMS invitation sender | 1 | 3 |
| Medical risk group | 1 | 3 |
| Vaccine frequency | 1 | 3 |
| Region of vaccine manufacturer | 1 | 3 |
| Mandatory testing at own cost if does not get vaccinated | 1 | 3 |
| Recommends | 1 | 3 |
| Time of Covid vaccination | 1 | 3 |
| **Low- and middle-income countries (N=24)** |  |  |
| Effectiveness | 10 | 42 |
| Safety | 4 | 17 |
| Cost | 3 | 13 |
| Brand | 1 | 4 |
| Potential capacity to spread the virus | 1 | 4 |
| Halal content | 1 | 4 |
| Mortality rate | 1 | 4 |
| Possible trends of the epidemic | 1 | 4 |
| Quarantine-free travel | 1 | 4 |
| The proportion of vaccinated friends/family | 1 | 4 |
