# Supplementary material for: Preferences for COVID-19 Vaccines: Systematic Literature Review of Discrete Choice Experiments
Source: JMIR Public Health Surveill. 2024 Jul 29;10:e56546. doi: 10.2196/56546 (PMC11319885; doi:10.2196/56546)
Supplement: Multimedia Appendix 6 [file publichealth_v10i1e56546_app6.docx]

**Multimedia Appendix 6. Preference for COVID-19 vaccines in the different study periods (N=53)**

| Most important attributes | Number | % |
| --- | --- | --- |
| **Before the pandemic wave(N=4)** |  |  |
| Effectiveness | 2 | 50 |
| Safety | 1 | 25 |
| Region of vaccine manufacturer | 1 | 25 |
| **During the pandemic wave(N=24)** |  |  |
| Effectiveness | 11 | 46 |
| Safety | 2 | 8 |
| Protection duration | 2 | 8 |
| Potential capacity to spread the virus | 1 | 4 |
| Recommenders | 1 | 4 |
| Halal content | 1 | 4 |
| Mandatory testing at own cost if does not get vaccinated | 1 | 4 |
| Medical risk group | 1 | 4 |
| Mortality risk | 1 | 4 |
| The proportion of Vaccinated friends/family | 1 | 4 |
| Time of Covid vaccination | 1 | 4 |
| Vaccine frequency | 1 | 4 |
| **After the pandemic wave(N=25)** |  |  |
| Effectiveness | 8 | 32 |
| Mortality risk | 5 | 20 |
| Safety | 4 | 16 |
| Cost | 3 | 12 |
| Brand | 1 | 4 |
| SMS invitation sender | 1 | 4 |
| Mortality rate | 1 | 4 |
| Possible trends of the epidemic | 1 | 4 |
| Quarantine-free travel | 1 | 4 |
