# Supplementary material for: Preferences for COVID-19 Vaccines: Systematic Literature Review of Discrete Choice Experiments
Source: JMIR Public Health Surveill. 2024 Jul 29;10:e56546. doi: 10.2196/56546 (PMC11319885; doi:10.2196/56546)
Supplement: Multimedia Appendix 7 [file publichealth_v10i1e56546_app7.docx]

**Multimedia Appendix 7. Assessment of 47 included studies quality using the Purpose, Respondents, Explanation, Findings, and Significance checklist**

| Title | Author, et.al., Year a | Purpose^b^ | Respondents^c^ | Explanation^e^ | Findings^f^ | Significance^g^ |
| --- | --- | --- | --- | --- | --- | --- |
| COVID-19 Vaccination Preferences Among Non-Chinese Migrants in Hong Kong: Discrete Choice Experiment | Asim et al,.2023 | 1 | 0 | 1 | 0 | 1 |
| COVID-19 vaccine preferences in India | Bansal et al,.2022 | 1 | 0 | 1 | 0 | 1 |
| Examination of Preferences for COVID-19 Vaccines in Hungary Based on Their Properties-Examining the Impact of Pandemic Awareness with a Hybrid Choice Approach | Blaga et al,.2023 | 1 | 0 | 1 | 0 | 1 |
| Preferences for a COVID-19 vaccine in Australia | Borriello et al,.2021 | 1 | 0 | 1 | 0 | 1 |
| Vaccination or NPI? A conjoint analysis of German citizens' preferences in the context of the COVID-19 pandemic | Bughin et al,.2023 | 1 | 0 | 1 | 0 | 1 |
| The COVID-19 vaccination decision-making preferences of elderly people: a discrete choice experiment | Chen et al,.2023 | 1 | 0 | 1 | 0 | 1 |
| Public preference and vaccination willingness for COVID-19 vaccine in China | Chen et al,.2021 | 1 | 0 | 1 | 0 | 1 |
| United States COVID-19 Vaccination Preferences (CVP): 2020 Hindsight | Craig,2021 | 1 | 0 | 1 | 0 | 1 |
| Public Preferences and Willingness to Pay for a COVID-19 Vaccine in Iran: A Discrete Choice Experiment | Darrudi et al,.2022 | 1 | 0 | 1 | 0 | 1 |
| A choice experiment assessment of stated early response to COVID-19 vaccines in the USA | Daziano,2022 | 1 | 0 | 1 | 1 | 1 |
| Quantifying healthcare and welfare sector workers' preferences around COVID-19 vaccination: a cross-sectional, single-profile discrete-choice experiment in France | Díaz Luévano et al,.2021 | 1 | 0 | 1 | 0 | 1 |
| Public preference for COVID-19 vaccines in China: A discrete choice experiment | Dong et al,.2020 | 1 | 0 | 1 | 0 | 1 |
| Acceptance of and Preference for COVID-19 Vaccination in India, the United Kingdom, Germany, Italy, and Spain: An International Cross-Sectional Study | Dong et al,.2022 | 1 | 0 | 1 | 1 | 1 |
| Factors Affecting Young Adults' Decision Making to Undergo COVID-19 Vaccination: A Patient Preference Study | Donin et al,.2022 | 1 | 1 | 1 | 0 | 1 |
| Preferences for COVID-19 vaccine distribution strategies in the US: A discrete choice survey | Eshun-Wilson et al,.2021 | 1 | 0 | 1 | 0 | 1 |
| Acceptance of and preference for COVID-19 vaccination in healthcare workers: a comparative analysis and discrete choice experiment | Fu et al,.2020 | 1 | 0 | 1 | 0 | 1 |
| COVID-19 Vaccination Preferences of University Students and Staff in Hong Kong | Fung et al,.2022 | 1 | 0 | 1 | 0 | 1 |
| South African University Staff and Students' Perspectives, Preferences, and Drivers of Hesitancy Regarding COVID-19 Vaccines: A Multi-Methods Study | George et al,.2022 | 1 | 0 | 1 | 0 | 1 |
| Preferences for COVID-19 Vaccination in People With Chronic Immune-Mediated Inflammatory Diseases | Hazlewood et al,.2023 | 1 | 0 | 1 | 1 | 1 |
| The path towards herd immunity: Predicting COVID-19 vaccination uptake through results from a stated choice study across six continents | Hess et al,.2022 | 1 | 0 | 1 | 0 | 0 |
| COVID-19 vaccine coverage, concerns, and preferences among Chinese ICU clinicians: a nationwide online survey | Huang et al,.2021 | 1 | 0 | 1 | 1 | 1 |
| Public preferences and willingness to accept a hypothetical vaccine to prevent a pandemic in Japan: a conjoint analysis | Igarashi et al,.2022 | 1 | 0 | 1 | 0 | 1 |
| Stated choice analysis of preferences for COVID-19 vaccines using the Choquet integral | Krueger et al,.2022 | 1 | 0 | 1 | 1 | 1 |
| Individual preferences for COVID-19 vaccination in China | Leng et al,.2021 | 1 | 0 | 1 | 0 | 1 |
| COVID-19 vaccine preferences among university students in Hong Kong: a discrete choice experiment | Li et al,.2021 | 1 | 0 | 1 | 0 | 1 |
| Understanding influencing attributes of COVID-19 vaccine preference and willingness-to-pay among Chinese and American middle-aged and elderly adults: A discrete choice experiment and propensity score matching study | Li et al,.2023 | 1 | 0 | 1 | 0 | 1 |
| A Comparison of Vaccine Hesitancy of COVID-19 Vaccination in China and the United States | Liu et al,.2021 | 1 | 0 | 1 | 0 | 1 |
| Rationing of a scarce life-saving resource: Public preferences for prioritizing COVID-19 vaccination | Luyten et al,.2022 | 1 | 0 | 1 | 0 | 1 |
| Efficacy or delivery? An online Discrete Choice Experiment to explore preferences for COVID-19 vaccines in the UK | McPhedran et al,.2021 | 1 | 0 | 1 | 1 | 1 |
| Location, location, location: a discrete choice experiment to inform COVID-19 vaccination programme delivery in the UK | McPhedran et al,.2022 | 1 | 0 | 1 | 1 | 1 |
| Public Preferences for a COVID-19 Vaccination Program in Quebec: A Discrete Choice Experiment | Morillon et al,.2022 | 1 | 0 | 1 | 0 | 1 |
| "Please, you go first!" preferences for a COVID-19 vaccine among adults in the Netherlands | Mouter et al,. 2022a | 1 | 0 | 1 | 0 | 1 |
| Public Preferences for Policies to Promote COVID-19 Vaccination Uptake: A Discrete Choice Experiment in The Netherlands | Mouter et al,. 2022b | 1 | 1 | 1 | 0 | 1 |
| Parental refusal and hesitancy of vaccinating children against COVID-19: Findings from a nationally representative sample of parents in the U.S | Panchalingam et al,.2022 | 1 | 0 | 1 | 0 | 1 |
| A Discrete Choice Analysis Comparing COVID-19 Vaccination Decisions for Children and Adults | Prosser et al,.2023 | 1 | 0 | 1 | 0 | 1 |
| COVID-19 vaccine hesitancy in a representative working-age population in France: a survey experiment based on vaccine characteristics | Schwarzinger et al,.2021 | 1 | 0 | 1 | 1 | 1 |
| How should COVID-19 vaccines be distributed between the Global North and South: a discrete choice experiment in six European countries | Steinert et al,.2022 | 1 | 0 | 1 | 0 | 1 |
| Malaysian public preferences and decision making for COVID-19 vaccination: A discrete choice experiment | Teh et al,.2022 | 1 | 0 | 1 | 0 | 1 |
| Preference and Willingness to Pay for the Regular COVID-19 Booster Shot in the Vietnamese Population: Theory-Driven Discrete Choice Experiment | Tran et al,.2023 | 1 | 0 | 1 | 0 | 0 |
| Regional differences in COVID-19 vaccine hesitancy in december 2020: A natural experiment in the French working-age population | Velardo et al,.2021 | 1 | 0 | 1 | 0 | 1 |
| Would COVID-19 vaccination willingness increase if mobile technologies prohibit unvaccinated individuals from public spaces? A nationwide discrete choice experiment from China | Wang et al,.2022 | 1 | 0 | 1 | 0 | 1 |
| Influence of Vaccination Characteristics on COVID-19 Vaccine Acceptance Among Working-Age People in Hong Kong, China: A Discrete Choice Experiment | Wang et al,.2021 | 1 | 0 | 1 | 0 | 1 |
| Impact of information framing and vaccination characteristics on parental COVID-19 vaccine acceptance for children: a discrete choice experiment | Wang et al,.2022 | 1 | 0 | 1 | 0 | 1 |
| Student COVID-19 vaccination preferences in China: A discrete choice experiment | Wang et al,. 2022a | 1 | 0 | 1 | 1 | 1 |
| Individual Preferences for COVID-19 Vaccination under the China's 2021 National Vaccination Policy: A Discrete Choice Experiment Study | Wang et al,. 2022b | 1 | 0 | 1 | 1 | 1 |
| Attribute nonattendance in COVID-19 vaccine choice: A discrete choice experiment based on Chinese public preference | Xiao et al,.2022 | 1 | 0 | 1 | 1 | 1 |

^a^ Two studies published by the same author in the same year were distinguished by ‘a’ ‘b’ labels.

^b^Purpose: Is the purpose of the study in relation to preferences clearly stated?

^c^Respondents: Are the responders similar to the non- responders?

^d^Explanation: Are methods of assessing preferences clearly explained?

^e^Findings: Were all respondents included in the reported findings and analysis of preference results?

^f^Significance: Were significance tests used to assess the preference results?
